# Concise total syntheses of phelligridins A, C, and D†

**DOI:** 10.1039/c8ra10346a

**Published:** 2019-03-05

**Authors:** Takayuki Ohyoshi, Keisuke Mitsugi, Tatsuya Higuma, Fumitaka Ichimura, Masahito Yoshida, Hideo Kigoshi

**Affiliations:** Department of Chemistry, Graduate School of Pure and Applied Sciences, University of Tsukuba 1-1-1 Tennodai Tsukuba 305-8571 Japan kigoshi@chem.tsukuba.ac.jp ohyoshi@chem.tsukuba.ac.jp

## Abstract

We have established a concise and scalable synthetic pathway for phelligridins A (1), C (2) and D (3). The synthetic highlights were Suzuki–Miyaura coupling and aldol-type condensation of α-pyrone. Phelligridin A was synthesized in four steps, while phelligridins C and D were each synthesized in six steps. Furthermore, we have revealed that the whole structure is essential for the cytotoxicity of phelligridins.

## Abstract 2

We have established a concise and scalable synthetic pathway for phelligridins A (1), C (2) and D (3) by using Suzuki–Miyamura coupling and aldol-type condensation as key steps.

## Graphical abstract

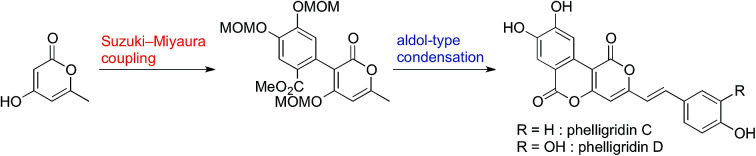

Phelligridin A (1) is a polyphenol isolated from the fruiting body of *Phellinus igniarius*, a fungus collected in Liaoning Province, China in 2003 (Fig. 1).^1^ A year later, analogs of 1, phelligridins C (2) and D (3), were also isolated from the fungus.^2^ Also in 2004, meshimakobnols A and B were independently isolated from the Japanese mushroom of the same genus (*Phellinus linteus*). Meshimakobnols A and B were found to be identical to 3 and 2, respectively.^3^ Structurally, they possess a tricyclic fused ring system including two adjacent α-pyrone rings. In addition, they inhibit the cell growth of a variety of cancer cells.^2,4^ However, the reported values of cytotoxicity against lung cancer A549 cells differed greatly. Furthermore, hispidin (4), a similar pyrone compound to phelligridin D (3), inhibits in a dose-dependent manner the activity of BACE 1, which produces amyloid β, a causative biomolecule of Alzheimer's disease.^5^ Recently, Shigemori and co-workers reported on the inhibitory effect of phelligridin D (3) on the aggregation of 42-mer amyloid β.^6^ However, the biological studies were limited to those of the natural products or their congeners. Because a structure–activity relationship study on phelligridins/meshimakobnols is expected to provide lead compounds for cancer and Alzheimer's disease, we decided to establish an adaptable synthetic route for their structure–activity relationships. Herein, we described a concise and scalable total synthesis of phelligridins A (1), C (2), and D (3) and their biological evaluation.

**Fig. 1:**
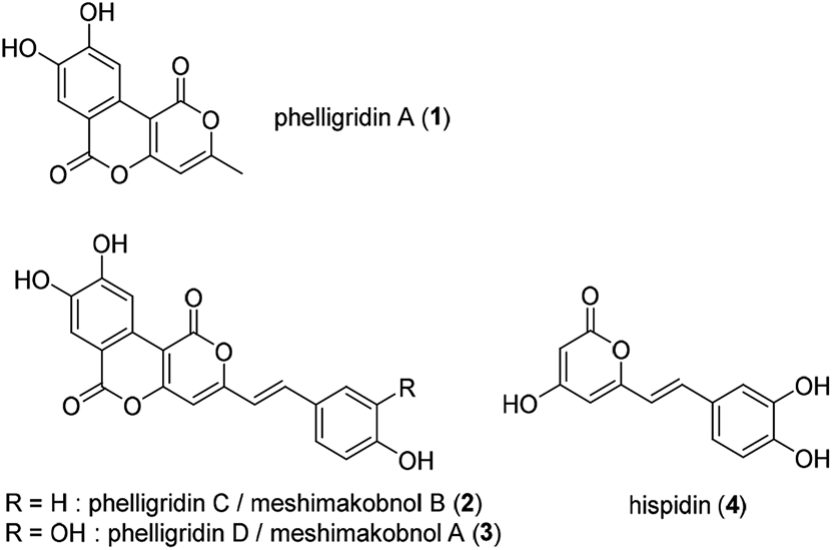
Structure of phelligridins A (1), C (2), D (3), and hispidin (4).

The retrosynthetic pathway of phelligridins is shown in Scheme 1. Phelligridins C (2) and D (3) would be derived from dimethylphelligridin A (6) by aldol-type condensation with the corresponding benzaldehyde. Dimethylphelligridin A (6) would be synthesized from boronate ester 7 and bromopyrone 8 by Suzuki–Miyaura coupling. In this synthetic plan, several analogs of the phelligridins can be synthesized by changing the two coupling partners of the aldol-type condensation and Suzuki–Miyaura coupling.

**Scheme 1:**
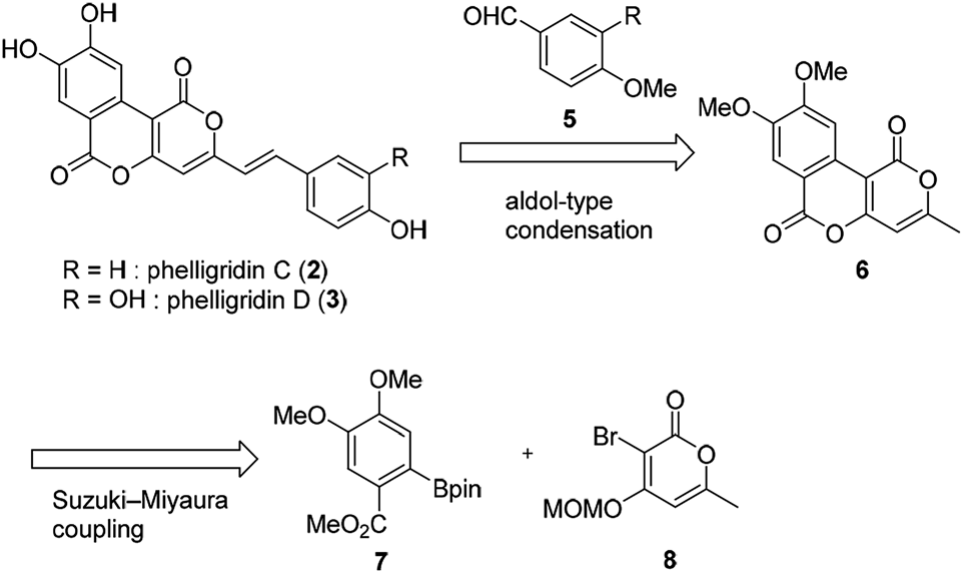
Retrosynthetic pathway of phelligridins.

Our synthesis of phelligridins started from the commercially available α-pyrone 9 (Scheme 2). Bromination^7^ and protection of 9 gave the bromopyrone 8, a precursor of Suzuki–Miyaura coupling. The coupling reaction between 8 and boronate ester 7 (ref. 8) followed by removal of the MOM group and concomitant lactonization gave dimethylphelligridin A (6) as a precursor of the aldol-type condensation. Next, we examined the synthesis of phelligridin D (3). The aldol-type reaction of dimethylphelligridin A (6) and aldehyde 5a was carried out under reported conditions, LHMDS/THF^9^ or ^*t*^BuOK/DMF.^10^ However, under these conditions, 6 was insoluble in solvent, and the reaction did not proceed.

**Scheme 2:**
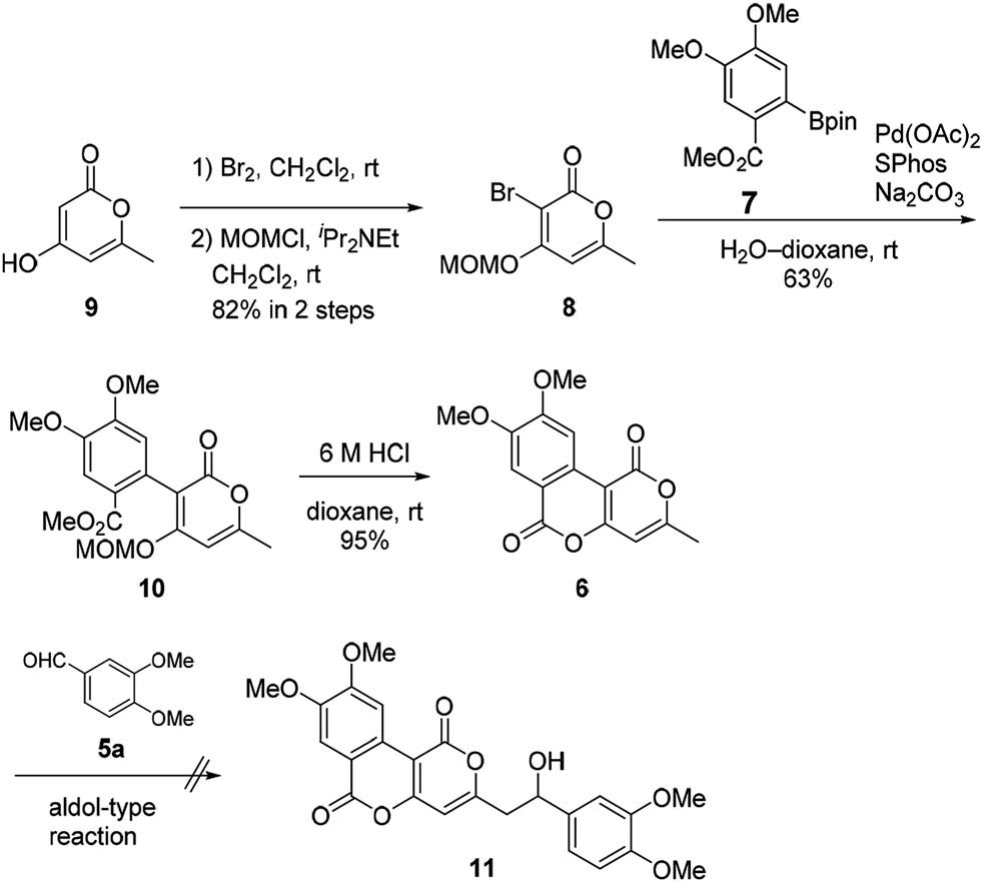
Synthesis of dimethylphelligridin A (6) and aldol-type reaction.

Therefore, we then tried aldol-type reaction with 10 (Scheme 3). The aldol reaction of 10 and 5a gave the aldol 12 in moderate yield. Subsequently, the aldol 12 was dehydrated *via* mesylation, and the MOM group was removed to obtain tetramethylphelligridin D (14). Finally, removal of the four methyl groups in 14 under HCl·py gave phelligridin D (3). Although we had achieved the total synthesis of 3, there are three problems needed to be solved to adapt the synthetic route to a scalable preparation. Firstly, the solubility of phelligridin D, as well as that of the synthetic intermediates from compound 10, was remarkably low in a variety of solvents. Therefore, there were restrictions on reaction conditions and purification methods. Secondly, isomerization of the trans olefin in 3 and 14 occurred easily by light. Finally, the demethylation of 14 required severe reaction conditions. All the aforementioned drawbacks resulted in a low overall yield for phelligridin D (3). In order to solve these three problems, we decided to protect the phenolic hydroxy groups as MOM ethers.

**Scheme 3:**
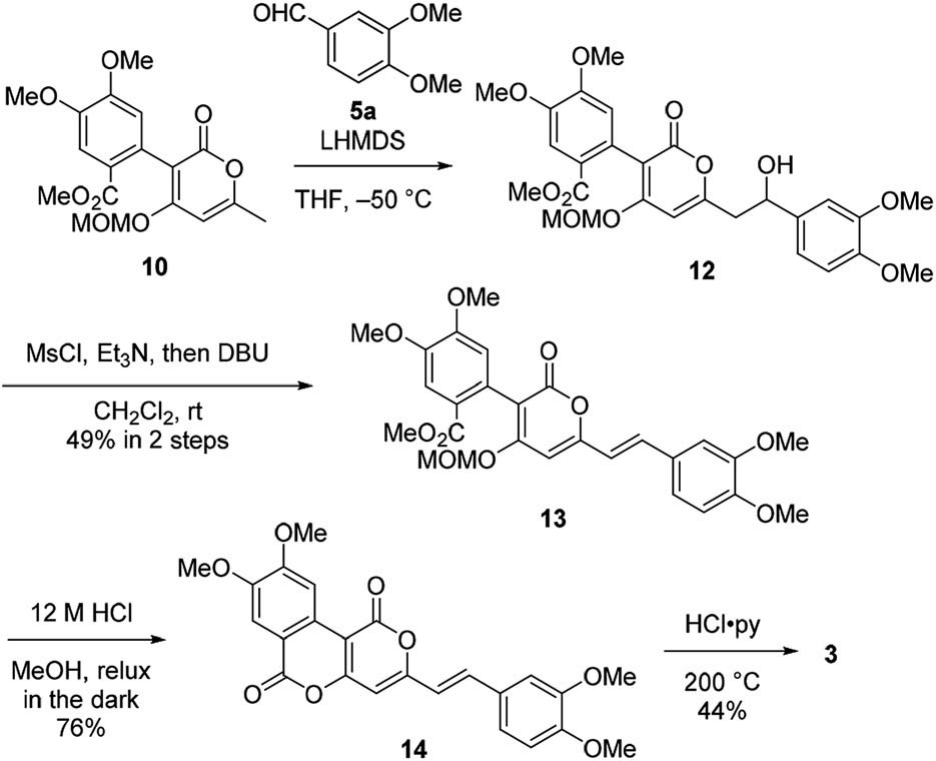
Total synthesis of phelligridin D (3).

Suzuki–Miyaura coupling of bromopyrone 8 and di-MOM boronate ester 15 gave the coupling compound 16 (Scheme 4). The aldol-type reaction with di-MOM aldehyde 17 and dehydration afforded 18. In this case, the aldol-type reaction of 16 proceeded in high yield presumably due to improved solubility of 16. Finally, phelligridin D (3) was synthesized by deprotection of five MOM groups under mild acidic conditions.

**Scheme 4:**
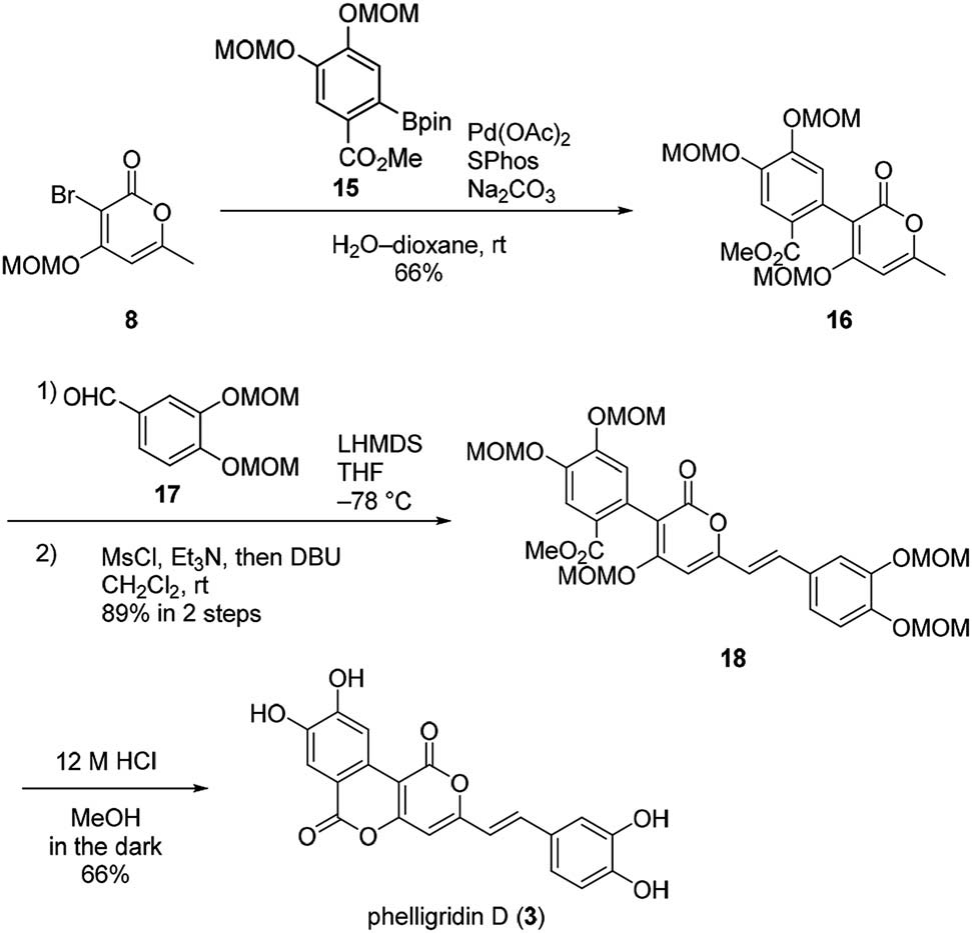
Concise total synthesis of phelligridin D (3).

Having established a synthetic route for phelligridin D (3), the total synthesis of phelligridin A (1) and C (2) was carried out using synthetic intermediate 16 (Scheme 5). Removal of the three MOM groups in 16 gave phelligridin A (1). In addition, phelligridin C (2) was synthesized in three steps from 16 by changing the aldehyde of the aldol-type reaction to 19.

**Scheme 5:**
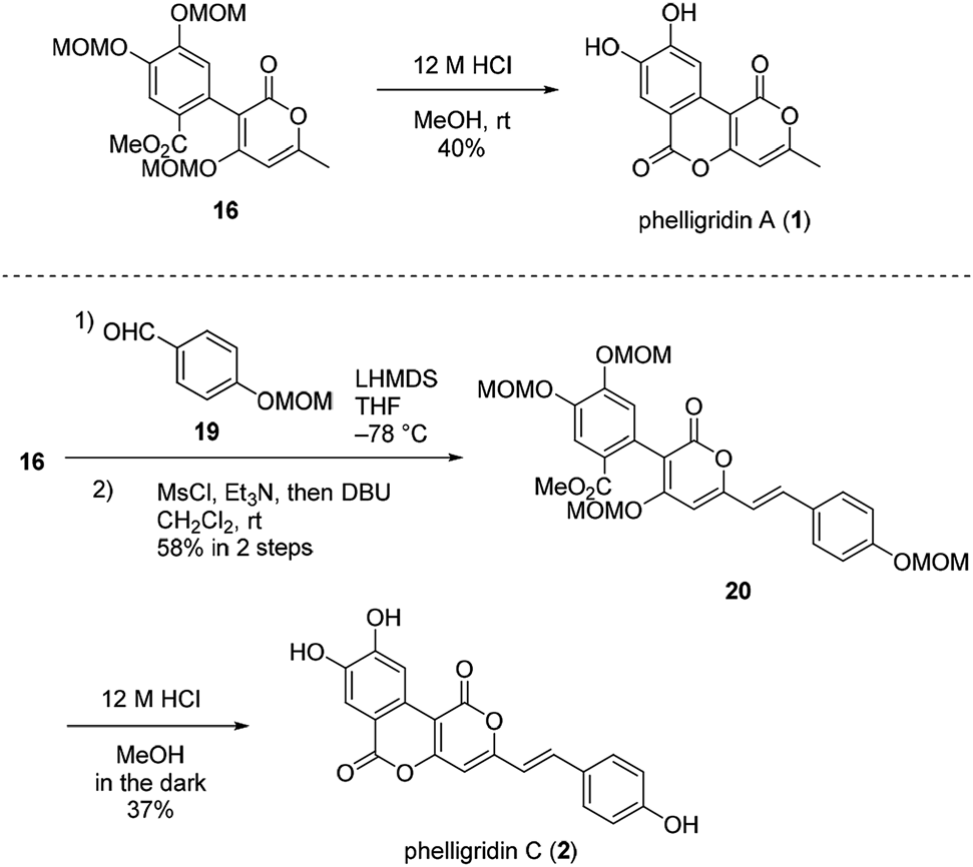
Total syntheses of phelligridins A (1), C (2).

With the synthetic samples in hand, the cytotoxicity against A549 cells and HeLa S3 cells of phelligridins were evaluated (Table 1). Phelligridin C (2) and D (3) showed cytotoxicity against A549 cells with IC_50_ of 1.6 μM and 1.1 μM, respectively. These values were closer to those reported by Nagatsu than by Shi.^11^ On the other hand, phelligridin A (1), a left segment of phelligridin C (2) and D (3), and hispidin (4), a right segment of phelligridin D (3), show no cytotoxicity. These results showed that the combination of left and right segments is essential for the cytotoxicity of the phelligridins. In addition, we evaluated cytotoxicity against human cervical cancer cells. In cytotoxicity against HeLa S3 cells, the same tendencies were observed as well.

**Table 1:** Cytotoxicity of phelligridins A, C, D, and hispidin

Cpd	A549 cells^a^ (μM)			HeLa S3 cells (μM)
	Syn.	Nat.		Syn.
	IC_50_	IC_50_	GI_50_	IC_50_
1	>100	>0.192^b^	—	>100
2	1.6	0.012^b^	15.0^c^	3.0
3	1.1	0.016^b^	22.6^c^	2.1
4	>100	—	—	>100

a See ESI.

b Ref. 2.

c Ref. 4.

## Conclusions

We have established a concise and scalable synthetic pathway for phelligridins A (1), C (2), and D (3). The synthetic highlights were Suzuki–Miyaura coupling and aldol-type condensation of α-pyrone. Phelligridin A was synthesized in four steps, while phelligridins C and D were each synthesized in six steps. Furthermore, we have investigated their cytotoxicity and revealed that the whole structure is essential for the cytotoxicity of phelligridins. A structure–activity relationships study based on this synthetic route is ongoing in our laboratory.

## Conflicts of interest

There are no conflicts to declare.

## Supplementary Material

RA-009-C8RA10346A-s001

## References

[cit1] Mo S.-Y., Yang Y.-C., He W.-Y., Shi J.-G. (2003). Chin. Chem. Lett..

[cit2] Mo S.-Y., Wang S.-J., Yang Y.-C., Chen X.-G., Shi J.-G. (2004). J. Nat. Prod..

[cit3] Nagatsu A., Itoh S., Tanaka R., Kato S., Haruna M., Kishimoto K., Hirayama H., Goda Y., Mizukami H., Ogihara Y. (2004). Tetrahedron Lett..

[cit4] Kojima K., Ohno T., Inoue M., Mizukami H., Nagatsu A. (2008). Chem. Pharm. Bull..

[cit5] Lee I.-K., Yun B.-S. (2011). J. Antibiot..

[cit6] Aihara Y., Kawaguchi A., Hanaki M., Murakami K., Irie K., Shigemori H. (2017). Heterocycles.

[cit7] March P. D., Mareno-Manas M., Pi R., Ripoll I., Sanchez-Ferrando F. (1985). J. Heterocycl. Chem..

[cit8] Genes C., Michel S., Tillequin F., Poree F. H. (2009). Tetrahedron.

[cit9] Preindl J., Schulthoff S., Wirts C., Lingnau J., Furstner A. (2017). Angew. Chem., Int. Ed..

[cit10] Kraus G. A., Wanninayake U. K. (2015). Tetrahedron Lett..

[cit11] Very recently, we got the personal communication from Shi that the unit of cytotoxicity was wrong in ref. 2 and the values of cytotoxicity should be given in μg mL^−1^. The revised values of cytotoxicity against A549 cells are as follows: 2, 4.4 μM (0.012 μg mL^−1^); 3, 6.1 μM (0.016 μg mL^−1^). Our results correspond to the revised values.

