# Supplementary material for: Concise total syntheses of phelligridins A, C, and D
Source: RSC Adv. 2019 Mar 5;9(13):7321–3. doi: 10.1039/c8ra10346a (PMC9061207; doi:10.1039/c8ra10346a)

Electronic Supplementary Material (ESI) for RSC Advances.
This journal is © The Royal Society of Chemistry 2019

Electronic Supplementary Information

Concise Total Syntheses of Phelligridins A, C, and D

Takayuki Ohyoshi,* Keisuke Mitsugi, Tatsuya Higuma, Fumitaka Ichimura, Masahito Yoshida, and Hideo Kigoshi*

Department of Chemistry, Graduate School of Pure and Applied Sciences, University of Tsukuba, Tennodai, Tsukuba 305-8571, Japan

Experimental Procedures and Spectral Date for All New Compounds.

General methods.

All moisture-sensitive reactions were performed under an atmosphere of argon or nitrogen, and the starting materials were azeotropically dried with benzene before use. Anhydrous MeOH, CH_2Cl_2, THF, toluene, DMSO, and pyridine were purchased from Kanto Chemical Co., Inc., or Wako Pure Chemical Industries Ltd., and used without further drying. TLC analyses were conducted on E. Merck precoated silica gel 60 F_{254} (0.25 mm layer thickness). Fuji Silysia silica gel BW-820MH (75–200 μm) and FL-60D (45–75 μm) were used for column chromatography. Optical rotations were measured with a JASCO DIP-370 polarimeter. Infrared (IR) spectra were recorded on a JASCO FT/IR-4100 instrument, and only selected peaks are reported in wavenumbers (cm^{-1}). ^1H and ^{13}C NMR spectra were recorded on a Bruker AVANCE 400 or a Bruker DPX 400 spectrometer. The ^1H and ^{13}C chemical shifts (δ) were referenciated with $CDCl_3$ (δ_H = 7.26 and δ_C = 77.0) or DMSO-d_6 (δ_H = 2.50 and δ_C = 39.5). *J* values are given in Hz. The following abbreviations are used for spin multiplicity: s = singlet, d = doublet, t = triplet, q = quartet, m = multiplet, and br = broad. High resolution ESI/TOF mass spectra were recorded on a JEOL AccuTOFCS JMS-T100CS spectrometer.

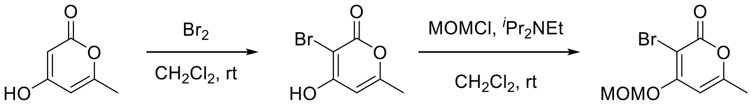

Bromopyrone 8.

To a stirred solution of 4-hydroxy-6-methyl-2-pyrone **9** (1.01 g, 7.99 mmol) in CH_2Cl_2 (80.0 mL) was added Br_2 (0.75 mL, 14.5 mmol) at room temperature. After being stirred at the same temperature for 5.5 h in the dark, the reaction mixture was concentrated. The crude bromide **9a** (1.63 g, 7.97 mmol) was used for the next reaction without further purification.

To a stirred solution of the crude bromide **9a** (1.63 g, 7.97 mmol) in CH_2Cl_2 (40.0 mL) were added ^{i}Pr$_2$NEt (4.20 mL, 24.1 mmol) and MOMCl (0.91 mL, 12.0 mmol) at 0 °C. The mixture was stirred at the room temperature for 13 h, diluted with saturated aqueous NH_4Cl (50 mL), and extracted with CH_2Cl_2 (3×50 mL). The combined extracts were dried (Na_2SO_4) and concentrated. The residual solid was purified by column chromatography on silica gel (50 g, hexane–EtOAc 1:1) to give bromopyrone **8** (1.63 g, 82% in 2 steps) as a colorless solid: m.p. 76.5–78.2 ºC; IR ($CHCl_3$) 3017, 2956, 2832, 1708, 1566, 1269, 1212, 1153,1088, 1003, 922, 798, 719 cm^{-1}; ^1H NMR (400 MHz, $CDCl_3$) δ 6.18 (s, 1H), 5.27 (s, 2H), 3.51 (s, 3H), 2.25 (s, 3H); ^{13}C NMR (100 MHz, $CDCl_3$) δ 165.2, 162.1, 160.9, 96.9, 94.7, 90.3, 57.1, 20.1; HRMS (ESI) *m/z* 270.9576 calcd for $C_8H_9BrNaO_4$ $[M+Na]^+$ 270.9582.

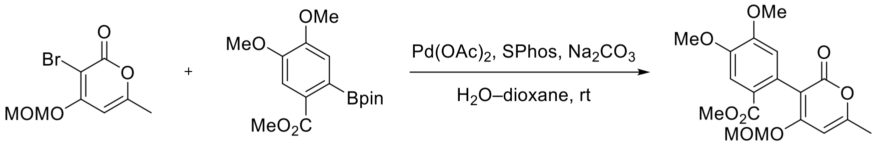

Coupling compound 10.

To the mixture of bromopyrone **8** (280 mg, 1.13 mmol), boronate ester **7** (435 mg, 1.35 mmol), $Pd(OAc)_2$ (27.9 mg, 0.124 mmol), SPhos (95.2 mg, 0.232 mmol), and Na_2CO_3 (369 mg, 3.48 mmol) was added degassed H_2O–dioxane (6:1) (11.1 mL). The mixture was stirred at the room temperature for 4 h, diluted with saturated aqueous NH_4Cl (12 mL), and extracted with EtOAc (3×20 mL). The combined extracts were dried (Na_2SO_4) and concentrated. The residual oil was purified by column chromatography on silica gel (25 g, hexane–EtOAc 1:1) to give coupling compound **10** (257 mg, 63%) as a colorless solid: m.p. 165.9–167.8 ºC; IR ($CHCl_3$) 3018, 1709, 1566, 1272, 1152, 1084, 1002, 924, 786 cm^{-1}; ^1H NMR (400 MHz, $CDCl_3$) δ 7.55 (s, 1H), 6.75 (s, 1H), 6.24 (q, J = 0.6 Hz, 1H), 5.07 (d, J = 7.0 Hz, 1H), 5.04 (d, J = 7.0 Hz, 1H), 3.93 (s, 3H), 3.89 (s, 3H), 3.75 (s, 3H), 3.36 (s, 3H), 2.31 (brs, 3H); ^{13}C NMR (100 MHz, $CDCl_3$) δ 166.5, 164.1, 163.3, 162.0, 151.8, 148.0, 126.9, 122.7, 114.3, 113.2, 107.6, 97.0, 93.9, 56.6, 56.0, 55.9, 51.8, 20.3; HRMS (ESI) 387.1075 *m/z,* calcd for $C_{18}H_{20}NaO_8$ $[M+Na]^+$ 387.1056.

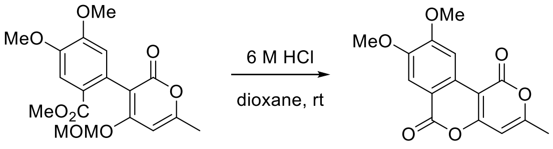

Dimethylphelligridin A (6).

To a stirred solution of coupling compound **10** (115 mg, 0.316 mmol) in dioxane (1.0 mL) was added 6 M HCl (1.0 mL, 6.0 mmol) at room temerature. After being stirred at the same temperature for 12 h, the reaction mixture was concentrated to give dimethylphelligridin A (**6**) (86.2 mg, 95%) as a colorless solid: m.p. 247.7–249.6 ºC; IR ($CHCl_3$) 3020, 1718, 1589, 1509, 1404, 1283, 1209, 1177, 1026, 822, 765, 731 cm^{-1}; ^1H NMR (400 MHz, $CDCl_3$) δ 8.55 (s, 1H), 7.68 (s, 1H), 6.24 (s, 1H), 4.08 (s, 3H), 4.00 (s, 3H), 2.38 (s, 3H); ^{13}C NMR (100 MHz, $CDCl_3$) δ 162.6, 161.4, 160.8, 159.4, 155.8, 149.9, 128.8, 112.9, 110.0, 106.7, 99.6, 99.0, 56.5, 56.2, 20.2; HRMS (ESI) 311.0561 *m/z,* calcd for $C_{15}H_{12}NaO_6$ $[M+Na]^+$ 311.0532.

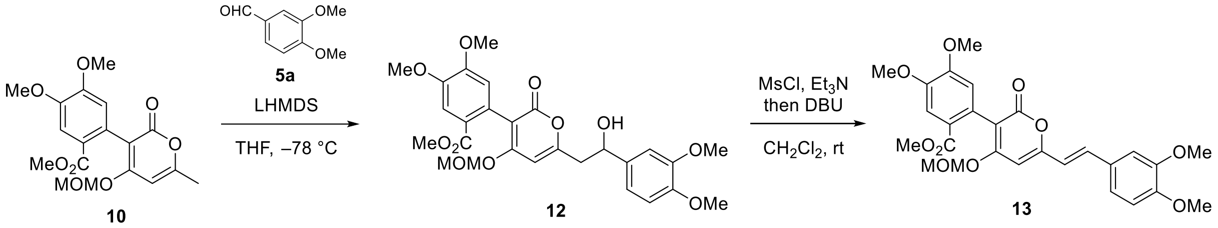

Compound 13.

To a stirred solution of coupling compound **10** (208 mg, 0.57 mmol) in THF (4.3 mL) was added LHMDS (1.0 M solution in THF, 1.8 mL, 1.8 mmol) at –50 °C. After stirring at same temperature for 1 h, a solution of aldehyde **5a** (111 mg, 0.67 mmol) in THF (0.70 mL) was added to the reaction mixture at –50 °C, and the mixture was stirred at same temperature for 3 h. The mixture was diluted with MeOH (5 ml) and saturated aqueous NH_4Cl (10 mL) and extracted with CH_2Cl_2 (3×10 mL). The combined extracts were dried (Na_2SO_4), filtered, and concentrated. The crude aldol **12** (296 mg) was used for the next reaction without further purification.

To a stirred solution of the crude aldol **12** (296 mg) in CH_2Cl_2 (2.8 mL) were added Et_3N (0.55 mL, 4.0 mmol) and MsCl (0.26 mL, 3.4 mmol) at 0 °C. After stirring for 1.5 h at same temperature, DBU (0.59 mL, 4.0 mmol) was added to the reaction mixture at 0 °C, and the mixture was stirred at same temperature for 1.5 h. The mixture was diluted with saturated aqueous NH_4Cl (10 mL) and extracted with CH_2Cl_2 (3×10 mL). The combined extracts were dried (Na_2SO_4), filtered, and concentrated. The residual solid was purified by column chromatography on silica gel (4 g, hexane–EtOAc 1:1) to give compound **13** (143 mg, 49% in 2 steps) as a yellow solid: m.p. 68.1–69.8 ºC; IR ($CHCl_3$) 3018, 1708, 1566, 1356, 1271, 1214, 1152, 1002, 788, 774, 721, 671 cm^{-1}; ^1H NMR (400 MHz, $CDCl_3$) δ 7.56 (s, 1H), 7.51 (d, J = 15.8 Hz, 1H), 7.09 (d, J = 8.3 Hz, 1H), 7.04 (s, 1H), 6.87 (d, J = 8.3 Hz, 1H), 6.80 (s, 1H), 6.55 (d, J = 15.8 Hz, 1H), 6.38 (s, 1H), 5.12 (d, J = 7.0 Hz, 1H), 5.08 (d, J = 7.0 Hz, 1H), 3.94 (s, 3H), 3.92 (s, 3H), 3.91 (s, 3H), 3.90 (s, 3H), 3.76 (s, 3H), 3.39 (s, 3H); ^{13}C NMR (100 MHz, $CDCl_3$) δ 166.7, 163.3, 163.2, 158.8, 151.7, 150.5, 149.2, 148.1, 135.7, 128.3, 126.9, 122.8, 121.6, 116.9, 114.3, 113.2, 111.2, 109.3, 108.8, 97.4, 94.1, 56.6, 55.99, 55.96, 55.9, 55.8, 51.8; HRMS (ESI) 535.1606 *m/z*, calcd for $C_{27}H_{28}NaO_{10}$ $[M+Na]^+$ 535.1580.

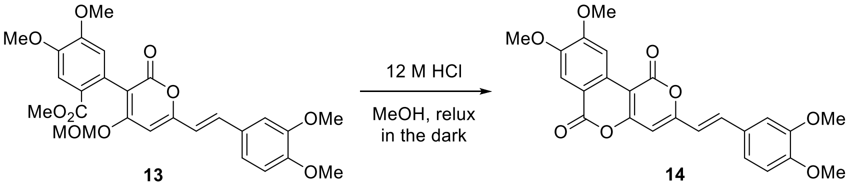

Tetramethylphelligridin D (14).

To a stirred solution of compound **13** (181 mg, 0.353 mmol) in MeOH (8.5 mL) was added 12 M HCl (2.0 mL, 24.0 mmol) at room temerature. After being stirred at reflux for 2 h in the dark, the reaction mixture was concentrated. The residual solid was washed with Et_2O to give tetramethylphelligridin D (**14**) (117 mg, 76%) as a yellow solid: m.p. 275.4–276.9 ºC; IR (KBr) 3180, 1715, 1514, 1369, 1285, 1225, 1156, 1034, 825, 637

cm^{-1}; ^{1}H NMR (400 MHz, $CDCl_3$) δ 8.58 (s, 1H), 7.69 (s, 1H), 7.55 (d, *J* = 15.8 Hz, 1H), 7.15 (d, *J* = 8.3 Hz, 1H), 7.08 (s, 1H), 6.89 (d, *J* = 8.3 Hz, 1H), 6.57 (d, *J* = 15.8 Hz, 1H), 6.35 (s, 1H), 4.11 (s, 3H), 4.01 (s, 3H), 3.96 (s, 3H), 3.93 (s, 3H) ; ^{13}C NMR (100 MHz, $CDCl_3$) δ 161.4, 160.1, 159.5, 159.0, 155.8, 151.0, 149.9, 149.4, 137.3, 129.1, 127.9, 122.3, 115.8, 112.9, 111.3, 110.1, 109.4, 106.8, 99.7, 99.0, 56.5, 56.3, 56.0, 56.0; HRMS (ESI) 459.1073 *m/z*, calcd for $C_{24}H_{20}NaO_8$ $[M+Na]^+$ 459.1056.

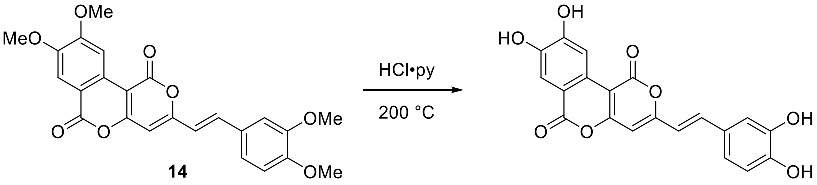

14
phelligridin D (**3**)

Phelligridin D (3).

The mixture of tetramethylphelligridin D (**14**) (11.9 mg, 27.3 μmol) and HCl•py (476 mg, 4.12 mmol) was stirred at the 200 °C for 3 h in the dark. The mixture was diluted with H_2O (10 mL) and extracted with CH_2Cl_2 (3×10 mL). The water layer was acidified with 12 M HCl (5 ml) and extracted with EtOAc (3×15 mL). The combined extracts were dried (Na_2SO_4), filtered, and concentrated to give phelligridin D (**3**) (4.6 mg, 44%) as a brown amorphous powder: IR (KBr) 3429, 3130, 1693, 1549, 1403, 1288, 1131, 781, 727, 683 cm^{-1}; ^{1}H NMR (400 MHz, $CDCl_3$) δ 10.75 (s, 1H), 10.10 (s, 1H), 9.58 (s, 1H), 9.17 (s, 1H), 8.33 (s, 1H), 7.51 (s, 1H), 7.28 (d, *J* = 15.3 Hz, 1H), 7.08 (s, 1H), 6.99 (d, *J* = 8.3 Hz, 1H), 6.78 (d, *J* = 15.3 Hz, 1H), 6.79 (d, *J* = 8.3 z, 1H), 6.71 (s, 1H); ^{13}C NMR (100 MHz, $CDCl_3$) δ 160.8, 159.5, 158.7, 158.4, 153.6, 147.9, 147.0, 145.7, 135.9, 127.0, 126.7, 120.9, 115.9, 115.5, 114.5, 114.1, 111.4, 110.5, 99.0, 98.8; HRMS (ESI) 379.0475 *m/z*, calcd for $C_{20}H_{11}O_8$ $[M–H]^-$ 379.0454.

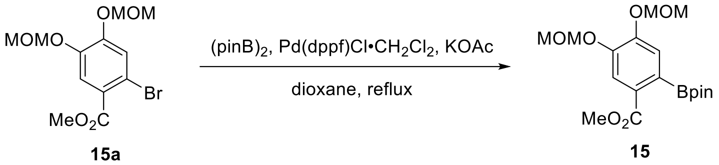

15a
15

Boronate ester 15.

The combined extracts were dried (Na_2SO_4), filtered, and concentrated.To the mixture of bromide **15a** (546 mg, 1.63 mmol), $(pinB)_2$ (680 mg, 2.68 mmol), Pd(dppf)•CH_2Cl_2 (87.1 mg, 0.107 mmol), and KOAc (664 mg, 6.77 mmol) was added degassed 1,4-dioxane (4.0 mL). The mixture was stirred at 80 ºC for 11 h, diluted with saturated aqueous NH_4Cl (15 mL), and extracted with EtOAc (3×15 mL). The combined extracts were dried (Na_2SO_4), and concentrated. The residual oil was purified by column chromatography on silica gel (50 g, hexane–EtOAc 7:1) to give boronate ester **15** (553 mg, 89%) as a colorless solid: m.p. 114.6–116.3 ºC; IR ($CHCl_3$) 3026, 3012, 1710, 1546, 1515, 1270, 1223, 1151, 1024, 787, 643, cm^{-1}; ^{1}H NMR (400 MHz,

$CDCl_3$) δ 7.71 (s, 1H), 7.19 (s, 1H), 5.28 (s, 2H), 5.25 (s, 2H), 3.88 (s, 3H), 3.50 (s, 3H), 3.49 (s, 3H), 1.40 (s, 12H); ^{13}C NMR (100 MHz, $CDCl_3$) δ 167.8, 150.5, 147.1, 127.7 (2C), 118.9, 117.1, 95.3, 95.1, 84.0 (2C), 56.4, 56.2, 52.2, 24.9 (4C); HRMS (ESI) 405.1710 *m/z*, calcd for $C_{18}H_{27}BNaO_8$ $[M+Na]^+$ 405.1697.

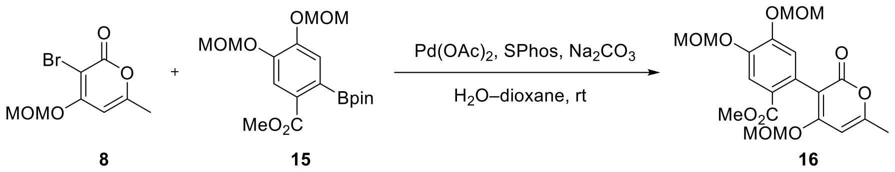

Coupling compound 16.

To the mixture of bromopyrone **8** (203 mg, 0.82 mmol), boronate ester **15** (473 mg, 1.24 mmol), $Pd(OAc)_2$ (41.8 mg, 0.19 mmol), SPhos (40.1 mg, 0.098 mmol), and Na_2CO_3 (422.8 mg, 3.99 mmol) was added degassed H_2O–dioxane (1:6) (17.5 mL). The mixture was stirred at room temperature for 5 h, diluted with saturated aqueous NH_4Cl (20 mL), and extracted with EtOAc (3×30 mL). The combined extracts were dried (Na_2SO_4), and concentrated. The residual oil was purified by column chromatography on silica gel (50 g, hexane–EtOAc 1:1) to give coupling compound **16** (228 mg, 66%) as a colorless solid: m.p. 95.2–96.8 ºC; IR ($CHCl_3$) 3019, 2961, 1711, 1546, 1509, 1269, 1153, 1070, 984, 925, 765 cm^{-1}; ^{1}H NMR (400 MHz, $CDCl_3$) δ 7.81 (s, 1H), 7.07 (s, 1H), 6.23 (s, 1H), 5.30 (d, *J* = 6.8 Hz, 1H), 5.28 (s, 2H), 5.21 (d, *J* = 6.8 Hz, 1H), 5.10 (d, *J* = 7.0 Hz, 1H), 5.02 (d, *J* = 7.0 Hz, 1H), 3.75 (s, 3H), 3.53 (s, 3H), 3.48 (s, 3H), 3.39 (s, 3H), 2.30 (s, 3H); ^{13}C NMR (100 MHz, $CDCl_3$) δ 166.4, 164.0, 163.2, 161.9, 150.1, 146.0, 128.1, 124.3, 119.1, 118.4, 107.4, 97.0, 95.3, 95.1, 93.9, 56.6, 56.3 (2C), 51.9, 20.3; HRMS (ESI) 447.1266 *m/z*, calcd for $C_{20}H_{24}NaO_{10}$ $[M+Na]^+$ 447.1267.

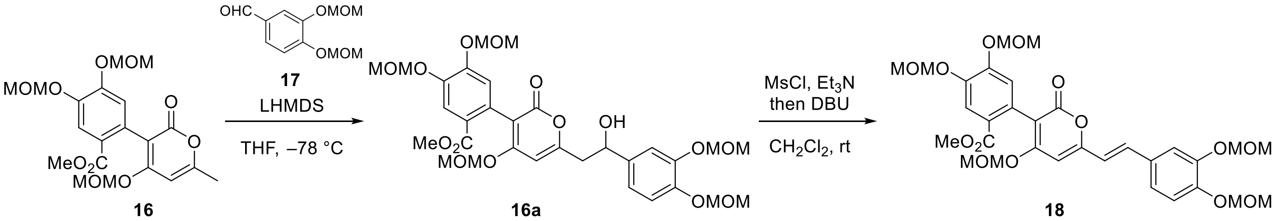

Compound 18.

To a stirred solution of coupling compound **16** (334 mg, 0.79 mmol) in THF (3.2 mL) was added LHMDS (1.0 M solution in THF, 1.60 mL, 1.60 mmol) at –78 °C. After stirring for 4 h at –78 ºC, a solution of aldehyde **17** (363 mg, 1.61 mmol) in THF (1.6 mL) was added to the reaction mixture at –78 °C, and the mixture was stirred at same temperature for 3.5 h. The mixture was diluted with MeOH (5 ml) and saturated aqueous NH_4Cl (10 mL) and extracted with CH_2Cl_2 (3×20 mL). The combined extracts were dried (Na_2SO_4), filtered, and concentrated. The crude aldol **16a** (783 mg) was used for the next reaction without further purification.

To a stirred solution of the crude aldol **16a** (783 mg) in CH_2Cl_2 (18 mL) were added Et_3N (1.3 mL, 9.33 mmol) and MsCl (0.49 mL, 6.32 mmol) at 0 °C. After stirring for 1.5 h at room temperature, DBU (1.4 mL, 9.38 mmol) was added to the reaction mixture at 0 °C, and the mixture was stirred at room temperature for 2.5 h. The mixture was diluted with saturated aqueous NH_4Cl (20 mL) and extracted with CH_2Cl_2 (3×40 mL). The combined extracts were dried (Na_2SO_4), filtered, and concentrated. The residual oil was purified by column chromatography on silica gel (50 g, hexane–EtOAc 1:1) to give compound **18** (444 mg, 89%) as a yellow oil: IR ($CHCl_3$) 3016, 1708, 1566, 1355, 1272, 1190, 1173, 1152, 1084, 1002, 795, 778, 770 cm^{-1}; ^1H NMR (400 MHz, $CDCl_3$) δ 7.83 (s, 1H), 7.49 (d, *J* = 15.9 Hz, 1H), 7.37 (d, *J* = 1.7 Hz, 1H), 7.17 (d, *J* = 8.3 Hz, 1H), 7.12 (dd, *J* = 8.3, 1.7 Hz, 1H), 7.13 (s, 1H), 6.55 (d, *J* = 15.9 Hz, 1H), 6.39 (s, 1H), 5.32 (d, *J* = 6.7 Hz, 1H), 5.30 (s, 2H), 5.27 (s, 2H), 5.27 (s, 2H), 5.23 (d, *J* = 6.7 Hz, 1H), 5.16 (d, *J* = 7.0 Hz, 1H), 5.08 (d, *J* = 7.0 Hz, 1H), 3.77 (s, 3H), 3.56 (s, 3H), 3.55 (s, 3H), 3.52 (s, 3H), 3.50 (s, 3H), 3.43 (s, 3H); ^{13}C NMR (100 MHz, $CDCl_3$) δ 166.5, 163.1, 158.6, 150.0, 148.5, 147.5, 146.1, 135.3, 130.0, 128.0, 124.5, 122.9 (2C), 119.1, 118.3, 117.6, 116.4, 114.9, 108.8, 97.7, 95.5, 95.3, 95.2, 95.1, 94.0, 56.6, 56.3, 56.3, 56.2 (2C), 51.9 ; HRMS (ESI) 655.2012 *m/z*, calcd for $C_{31}H_{36}NaO_{14}$ $[M+Na]^+$ 655.2003.

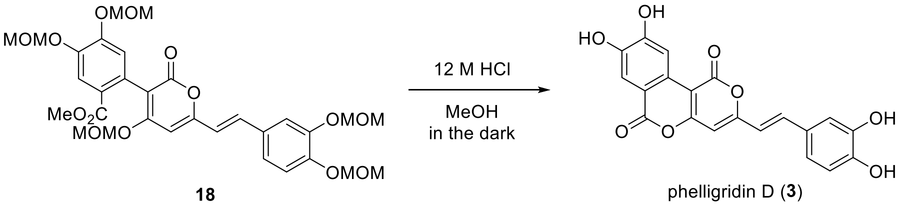

Phelligridin D (3).

To a stirred solution of compound **18** (444 mg, 0.70 mmol) in MeOH (150 mL) was added 12 M HCl (15.0 mL, 180 mmol) at room temerature. After being stirred at reflux for 5 h in the dark, the reaction mixture was concentrated. The residual solid was washed with Et_2O to give phelligridin D (**3**) (175 mg, 66%) as a brown amorphous powder. The spectra data were in full agreement with described above.

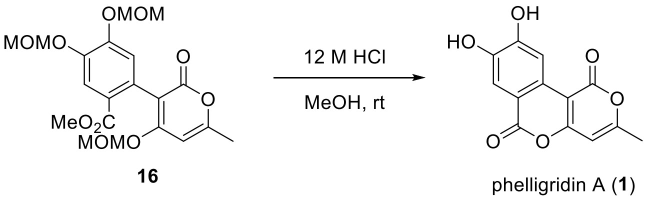

Phelligridin A (1).

To a stirred solution of compound **16** (8.9 mg, 21 μmol) in MeOH (1.0 mL) was added 12 M HCl (0.10 mL, 1.2 mmol) at room temerature. After being stirred at room temperature for 24 h , the reaction mixture was concentrated. The residual solid was washed with Et_2O to give phelligridin A (**1**) (2.1 mg, 40%) as a colorless solid: m.p. >300 ºC; IR (KBr) 3410, 3139, 1677, 1590, 1411, 1289, 1109, 1007, 899, 816, 756, 721

cm^{-1}; ^{1}H NMR (400 MHz, DMSO-d_6) δ 10.7 (s, 1H), 10.1 (s, 1H), 8.29 (s, 1H), 7.51 (s, 1H), 6.57 (s, 1H), 2.31 (s, 3H); ^{13}C NMR (100 MHz, DMSO-d_6) δ 162.3, 160.7, 160.2, 158.5, 153.5, 146.8, 126.6, 114.3, 111.2, 110.3, 99.2, 97.9, 19.3; HRMS (ESI) 259.0250 *m/z*, calcd for $C_{13}H_7O_6$ [M–H]$^-$ 259.0243.

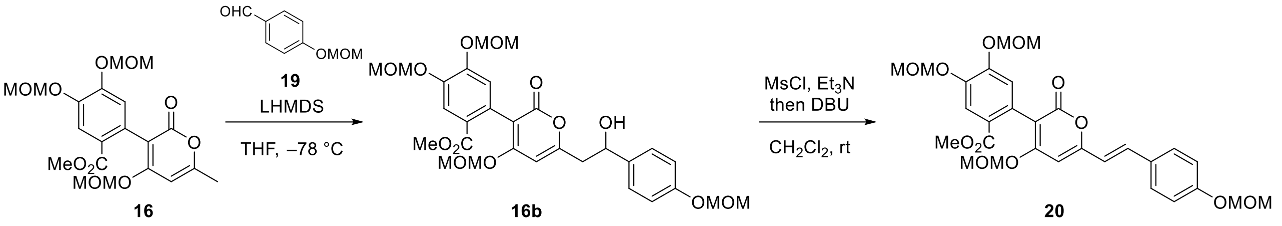

Compound 20.

To a stirred solution of coupling compound **16** (88.3 mg, 0.21 mmol) in THF (1.0 mL) was added LHMDS (1.0 M solution in THF, 0.42 mL, 0.42 mmol) at –78 °C. After stirring for 1 h at same temperature, a solution of aldehyde **17** (96.2 mg, 0.43 mmol) in THF (0.40 mL) was added to the reaction mixture at –78 °C, and the mixture was stirred at same temperature for 4 h. The mixture was diluted with MeOH (2 ml) and saturated aqueous NH_4Cl (10 mL) and extracted with CH_2Cl_2 (3×20 mL). The combined extracts were dried (Na_2SO_4), filtered, and concentrated. The crude aldol **16b** (220 mg) was used for the next reaction without further purification.

To a stirred solution of the crude aldol **16b** (220 mg) in CH_2Cl_2 (4.2 mL) were added Et_3N (0.36 mL, 2.6 mmol) and MsCl (0.14 mL, 1.81 mmol) at 0 °C. After stirring for 1.5 h at room temperature, DBU (0.40 mL, 2.68 mmol) was added to the reaction mixture at 0 °C, and the mixture was stirred at room temperature for 7 h. The mixture was diluted with saturated aqueous NH_4Cl (10 mL) and extracted with CH_2Cl_2 (3×10 mL). The combined extracts were dried (Na_2SO_4), filtered, and concentrated. The residual oil was purified by column chromatography on silica gel (20 g, hexane–EtOAc 1:1) to give compound **20** (69.8 mg, 58% in 2 steps) as a yellow oil: IR ($CHCl_3$) 3018, 1707, 1600, 1570, 1439, 1346, 1274, 1142, 1085, 978, 931, 857, 643 cm^{-1}; ^{1}H NMR (400 MHz, $CDCl_3$) δ 7.83 (s, 1H), 7.52 (d, J = 15.9 Hz, 1H), 7.46 (d, J = 8.7 Hz, 2H), 7.12 (s, 1H), 7.05 (d, J = 8.7 Hz, 2H), 6.55 (d, J = 15.9 Hz, 1H), 6.37 (s, 1H), 5.31 (d, J = 6.8 Hz, 1H), 5.30 (s, 2H), 5.23 (d, J = 6.8 Hz, 1H), 5.20 (s, 2H), 5.16 (d, J = 7.0 Hz, 1H), 5.07 (d, J = 7.0 Hz, 1H), 3.77 (s, 3H), 3.54 (s, 3H), 3.49 (s, 3H), 3.49 (s, 3H), 3.42 (s, 3H); ^{13}C NMR (100 MHz, $CDCl_3$) δ 166.5, 163.2, 158.8, 158.3, 150.0, 146.1, 135.4, 129.3, 128.9 (2C), 128.1, 124.5, 119.1, 118.3, 117.2, 116.6 (2C), 108.7, 97.5 (2C), 95.4, 95.1, 94.3, 94.0, 56.6, 56.3 (2C), 56.1, 51.9 ; HRMS (ESI) 595.1773 *m/z*, calcd for $C_{29}H_{32}NaO_{12}$ [M+Na]$^+$ 595.1791.

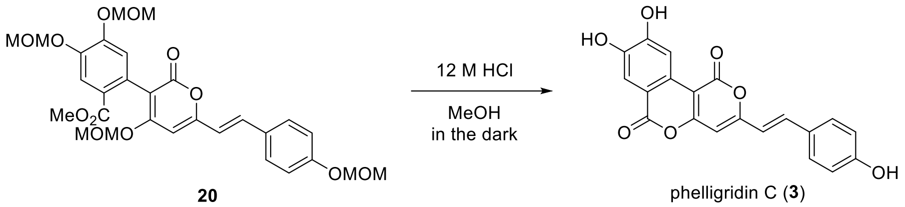

Phelligridin C (2).

To a stirred solution of compound **20** (69.8 mg, 0.12 mmol) in MeOH (26 mL) was added 12 M HCl (2.6 mL, 31 mmol) at room temerature. After being stirred at reflux for 5 h in the dark, the reaction mixture was concentrated. The residual solid was washed with Et_2O to give phelligridin C (**2**) (16.1 mg, 37%) as a brown amorphous powder : IR (KBr) 3432, 3282, 1668, 1555, 1405, 1280, 1137, 761, 721, 684 cm^{-1}; ^{1}H NMR (400 MHz, DMSO-d_6) δ 10.7 (s, 1H), 10.1 (s, 1H), 9.97 (s, 1H), 8.29 (s, 1H), 7.49 (d, J = 8.2 Hz, 2H), 7.48 (s, 1H), 7.32 (d, J = 16.0 Hz, 1H), 6.84 (d, J = 16.0 Hz, 1H), 6.78 (d, J = 8.2 Hz, 2H), 6.63 (s, 1H); ^{13}C NMR (100 MHz, DMSO-d_6) δ 160.7, 159.5, 159.3, 158.7, 158.4, 153.6, 147.0, 135.5, 129.6, 127.0 (2C), 126.2, 115.9 (2C), 115.6, 114.5, 111.4, 110.5, 99.0, 98.8; HRMS (ESI) 363.0493 *m/z*, calcd for $C_{20}H_{11}O_7$ $[M–H]^-$ 363.0505.

Cytotoxic activity

Stock cultures of cancer cells (HeLa S3, A549 cells: A549 cells were kindly provided by Prof. Hiroshi Nakagawa at the Department of Applied Biological Chemistry in Chubu University) were maintained in Dulbecco's Modified Eagle Medium containing 10% fetal bovine serum and 100 μg/mL of penicillin, and 100 μg/mL streptomycin at 37 °C under 5% CO_2. For the purpose of the experiment, 5×10^3 cells suspended in 100 μL of medium per well were plated in 96-well plate, and incubated at 37 °C under 5% CO_2. After incubation for 24 h, a solution of compound in DMSO (1 μL, concentration: 0.001, 0.01, 0.1, 1, 10 mM, respectively) was added to the above-mentioned well, resulting in various concentrations of the compound (0.01, 0.1, 1, 10, 100 μM) or solvent control (1% DMSO). After incubation for 96 h under the same conditions, 5 μL of WST-8 reagent solution was added to the cell culture, and the cell culture was further incubated for 2 h. Colorimetric determination of WST-8 was conducted at 450 nm with an optical reference wavelength at 595 nm using a microplate reader. The absorbance obtained upon the addition of the vehicle was considered as 100%. Data are expressed from the dose-response curve at three independent experiments. The cytotoxic effects of each compound were obtained as IC_{50} values calculated by probit analysis using the PriProbit 1.63 software.

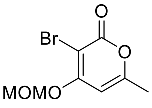
O
Br
O
MOMO

8

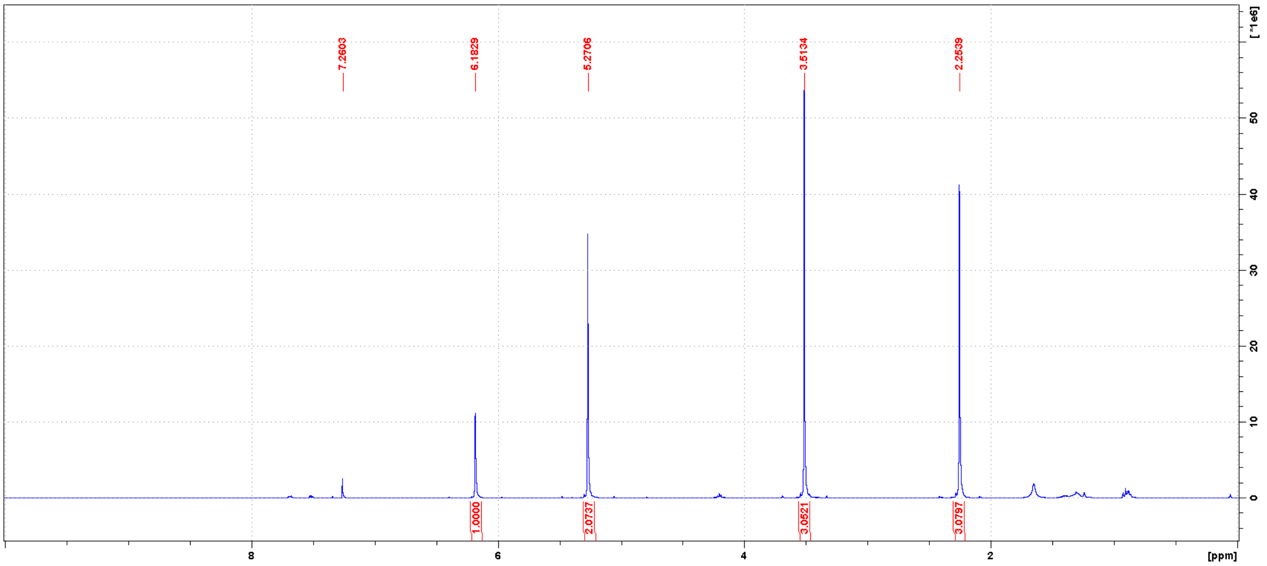
7.2603
6.1829
5.2706
3.5134
2.2539
1.0000
2.0737
3.0521
3.0797
[ppm]
[*1e6]

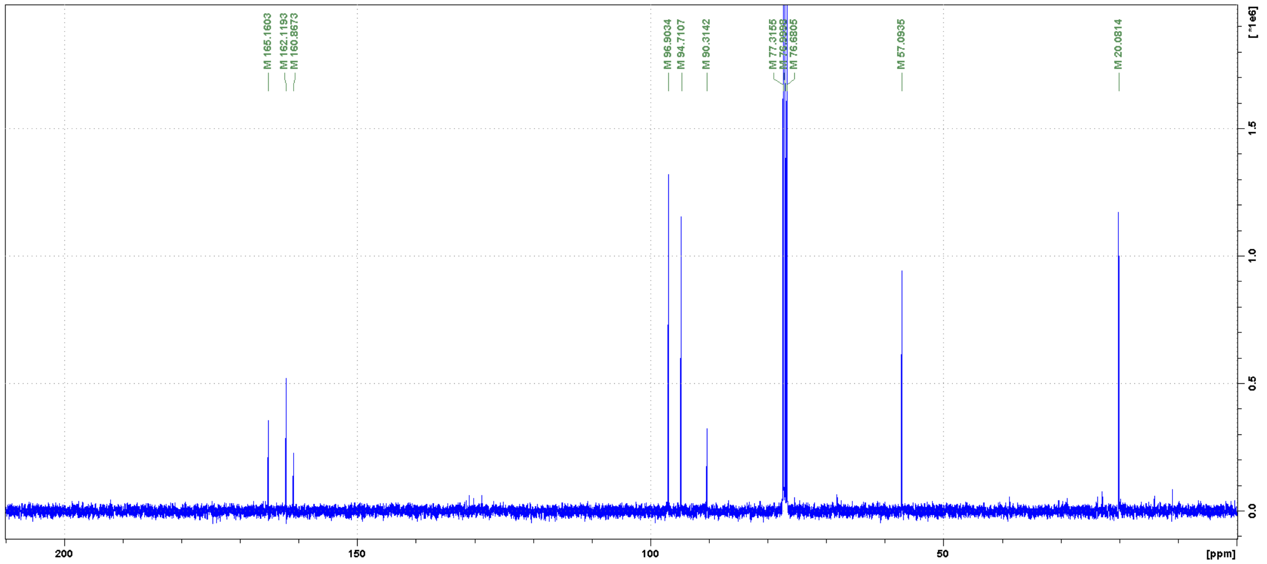
M 165.1603
M 162.1193
M 160.8673
M 96.9034
M 94.7107
M 90.3142
M 77.3165
M 76.6805
M 57.0935
M 20.0814
[ppm]
[*1e6]

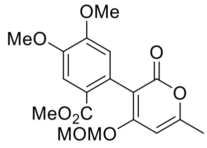

OMe
MeO
O
O
MeO2C
MOMO

10

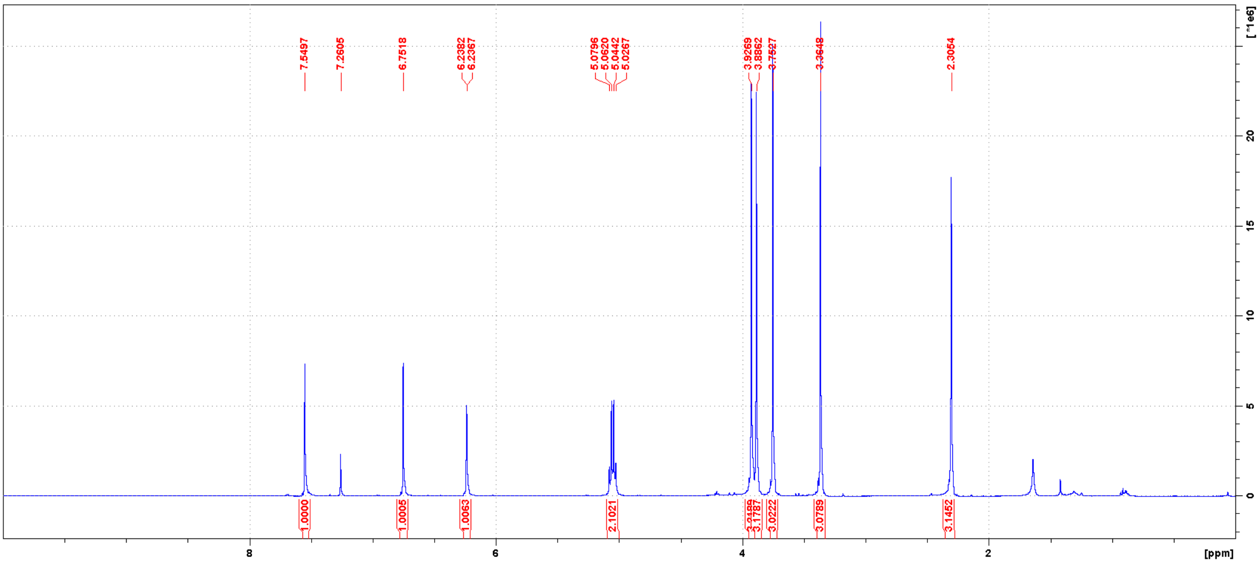

7.5497
7.2605
6.7518
6.2382
6.2367
5.0796
5.0620
5.0442
5.0267
3.9269
3.8862
3.7627
3.3648
2.3054
1.0000
1.0005
1.0063
2.1021
3.1787
3.0222
3.0789
3.1452
[*1e6]
[ppm]

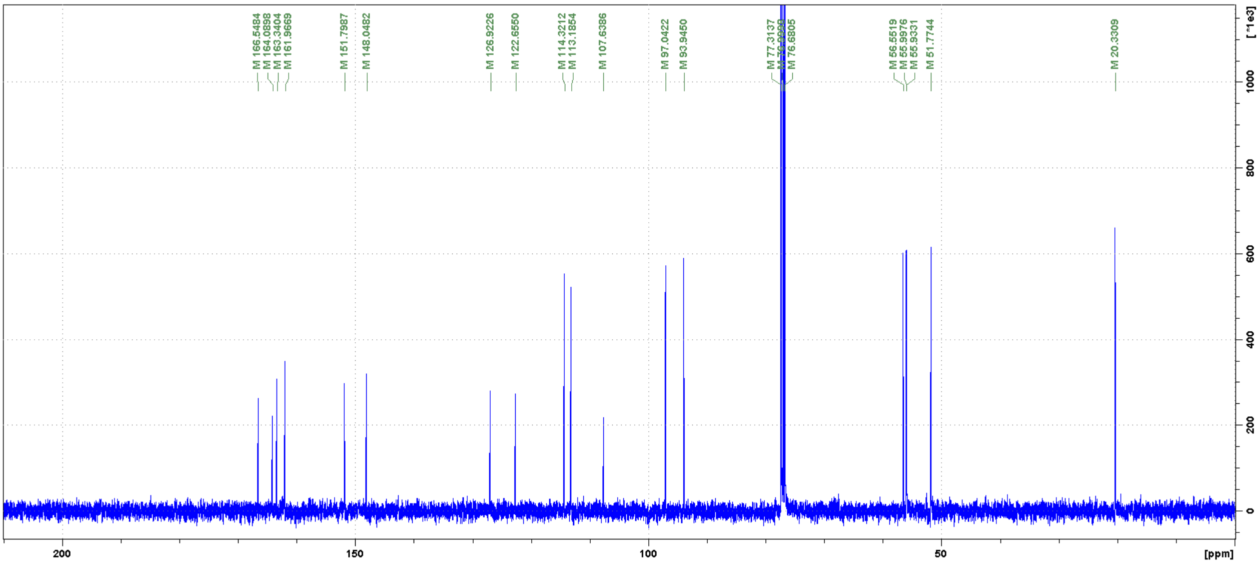

M 166.5484
M 164.0898
M 163.3404
M 161.9669
M 151.7987
M 148.0482
M 126.9226
M 122.6550
M 114.3212
M 113.1854
M 107.6386
M 97.0422
M 93.9450
M 77.3137
M 76.6805
M 56.5519
M 55.9976
M 55.9331
M 51.7744
M 20.3309
[*1e3]
[ppm]

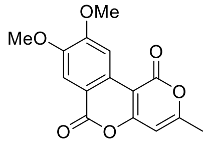

6

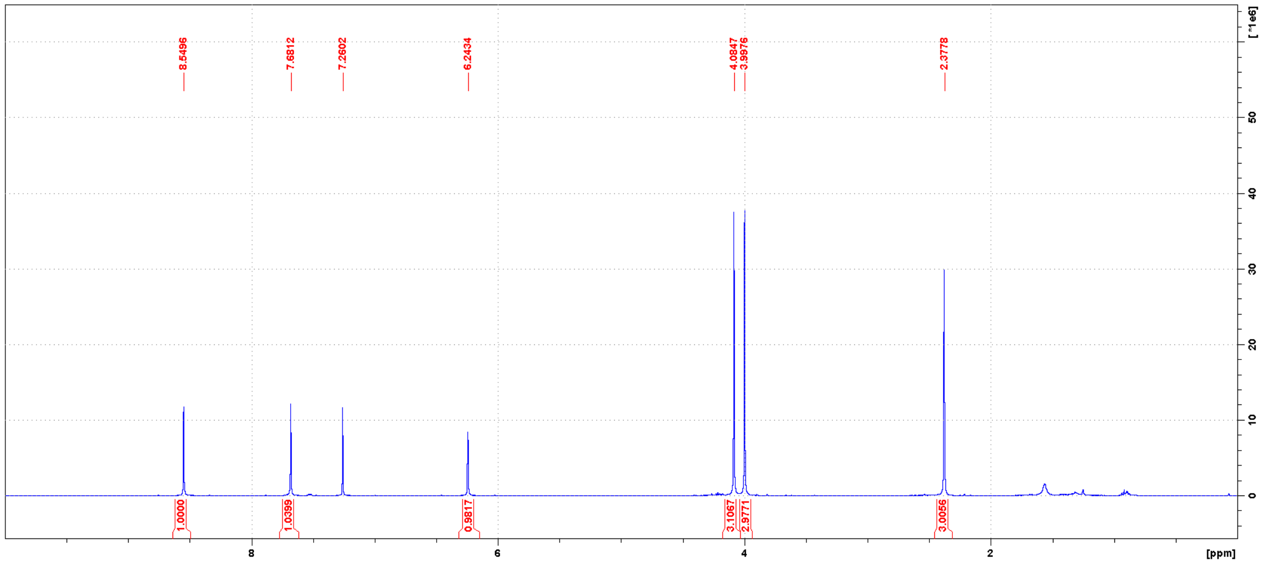

8.5496
7.6812
7.2602
6.2434
4.0847
3.9976
2.3778
1.0000
1.0399
0.9817
3.1067
2.9771
3.0056
[ppm]
[*1e6]

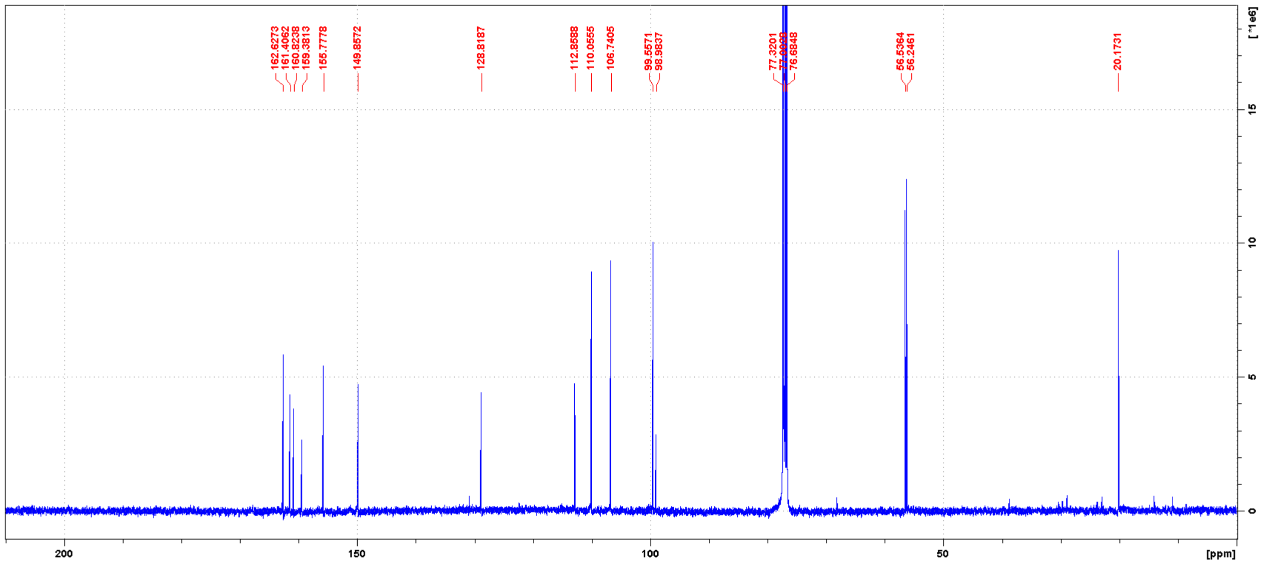

162.6273
161.4062
160.8238
159.3813
155.7778
149.8572
128.8187
112.8588
110.0555
106.7405
99.5571
98.9837
77.3201
76.6848
56.5364
56.2461
20.1731
[ppm]
[*1e6]

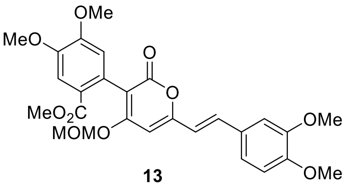

13

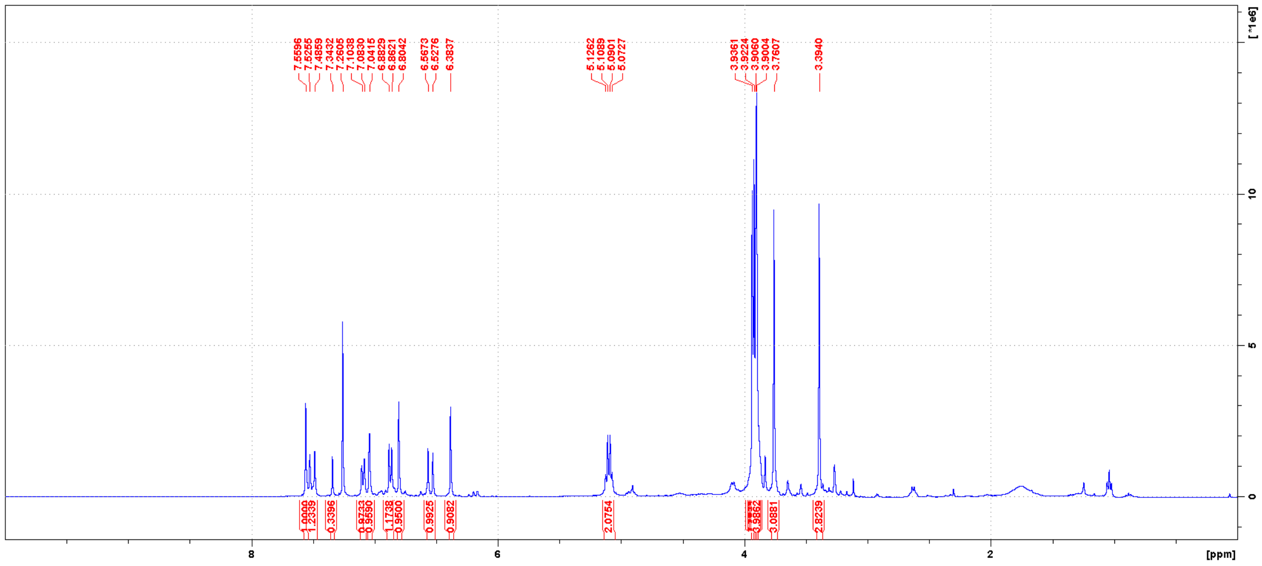

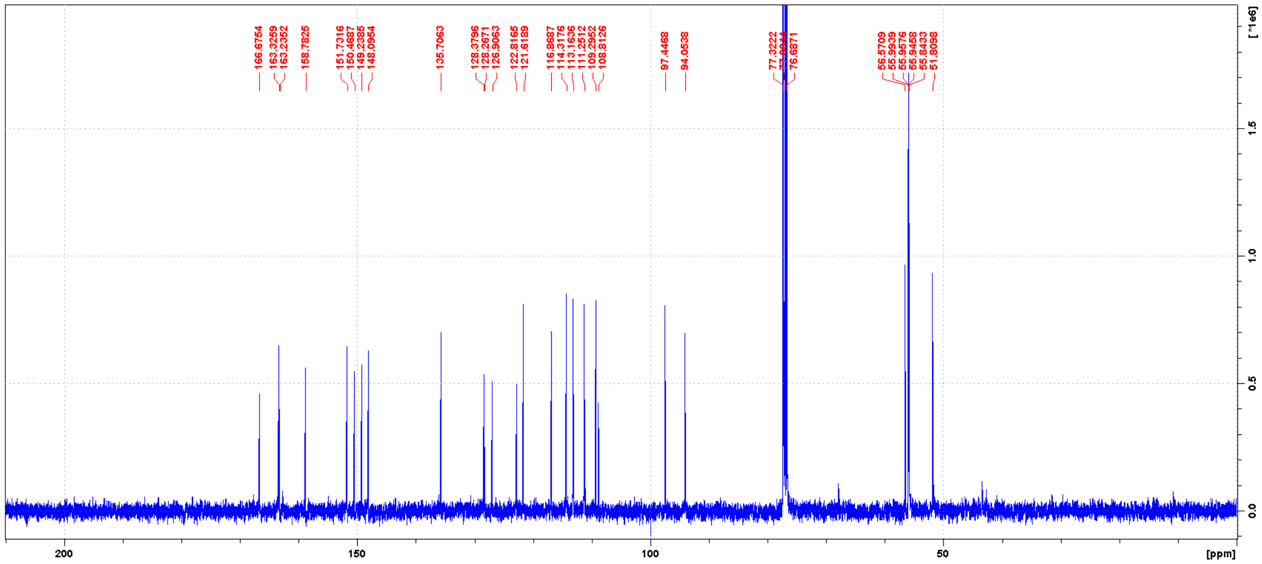

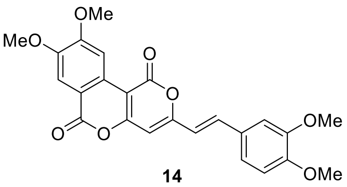

14

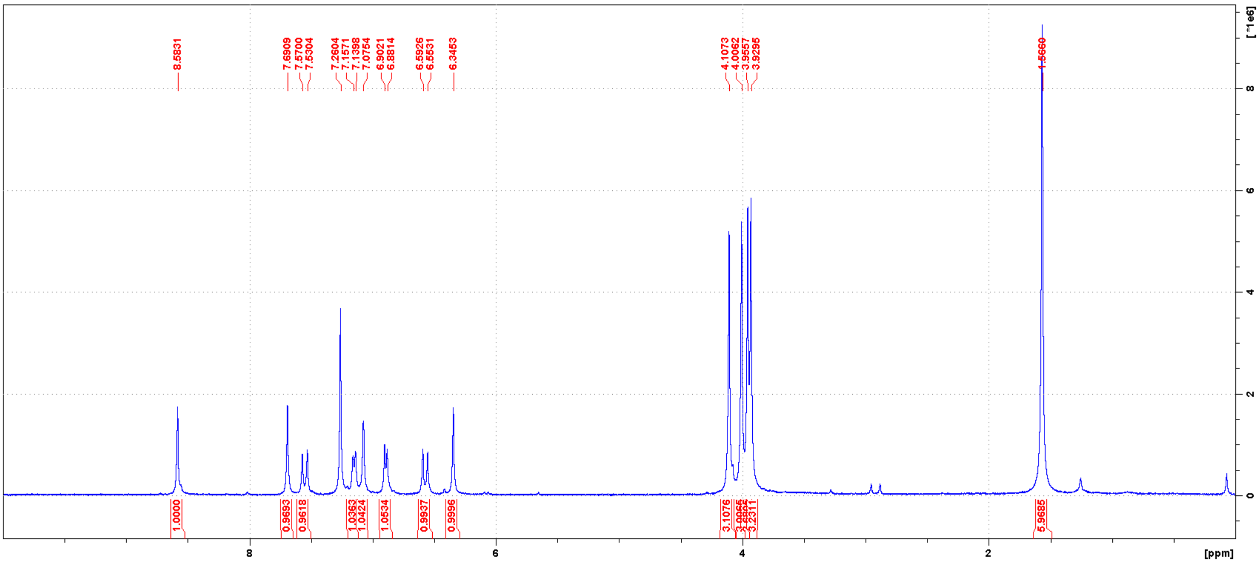

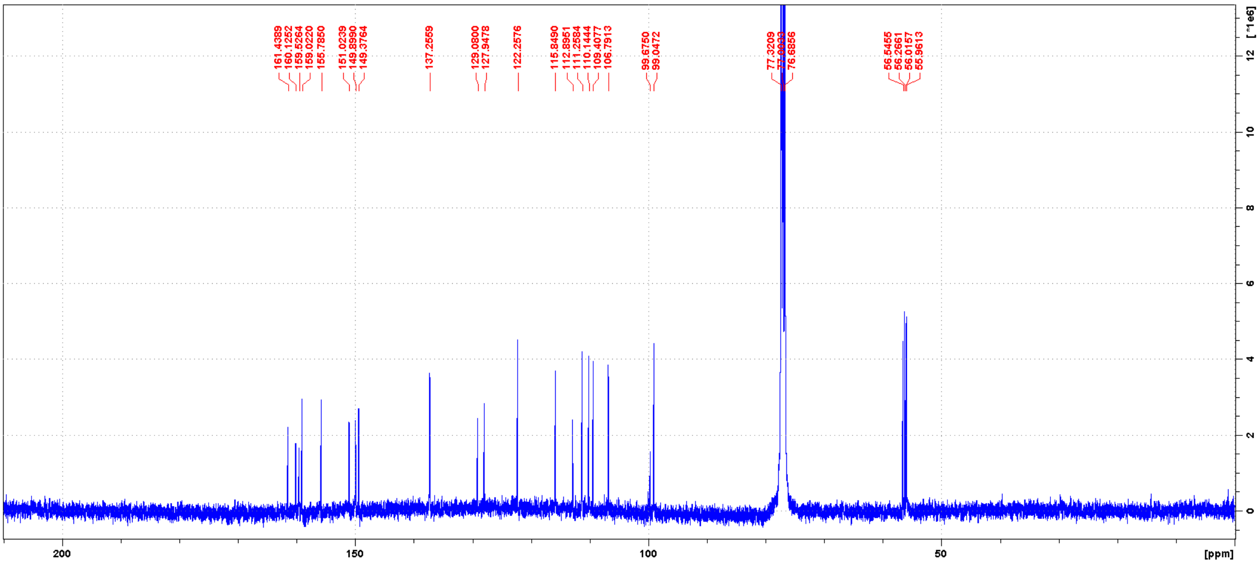

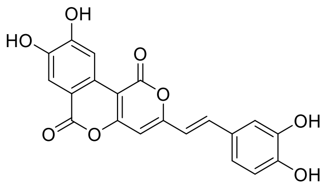

phelligridin D (**3**)

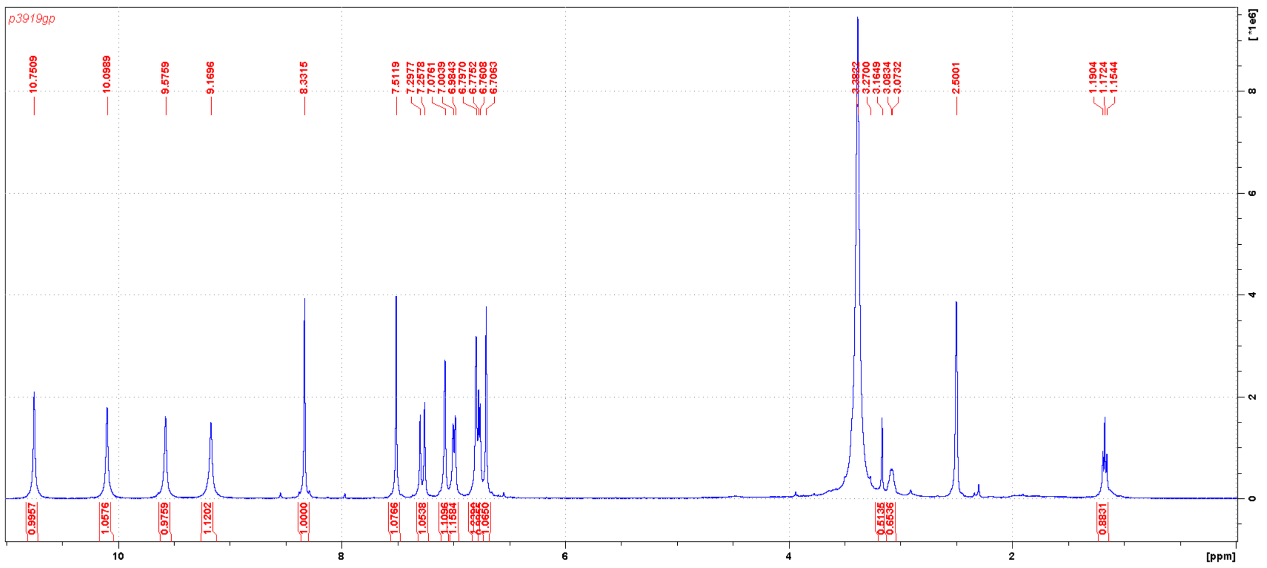

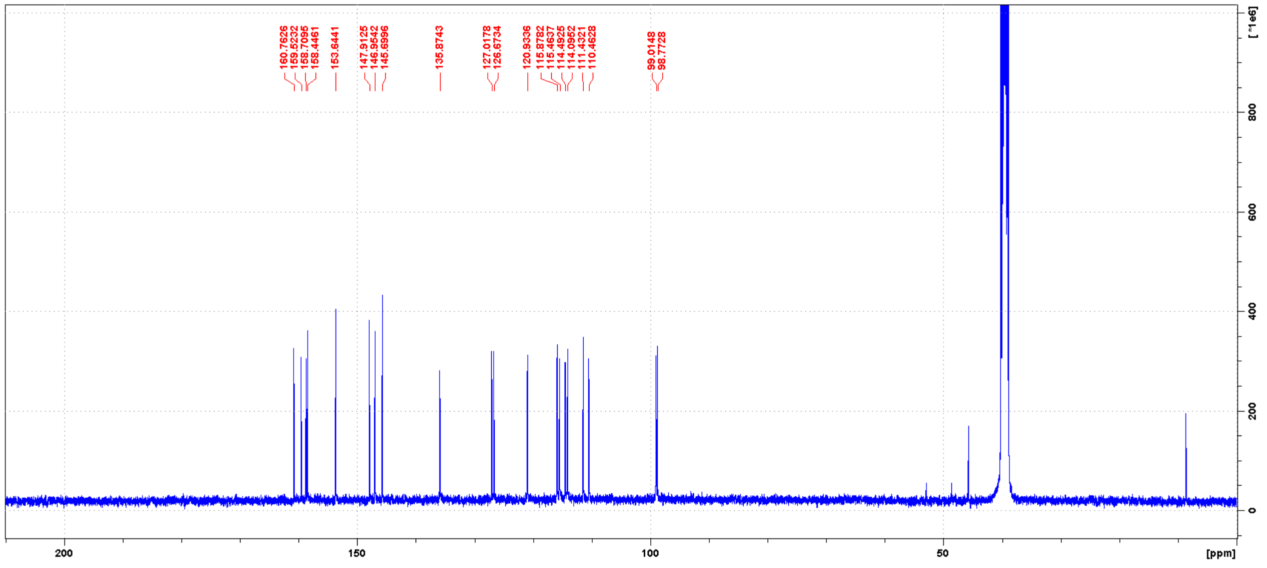

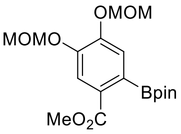

15

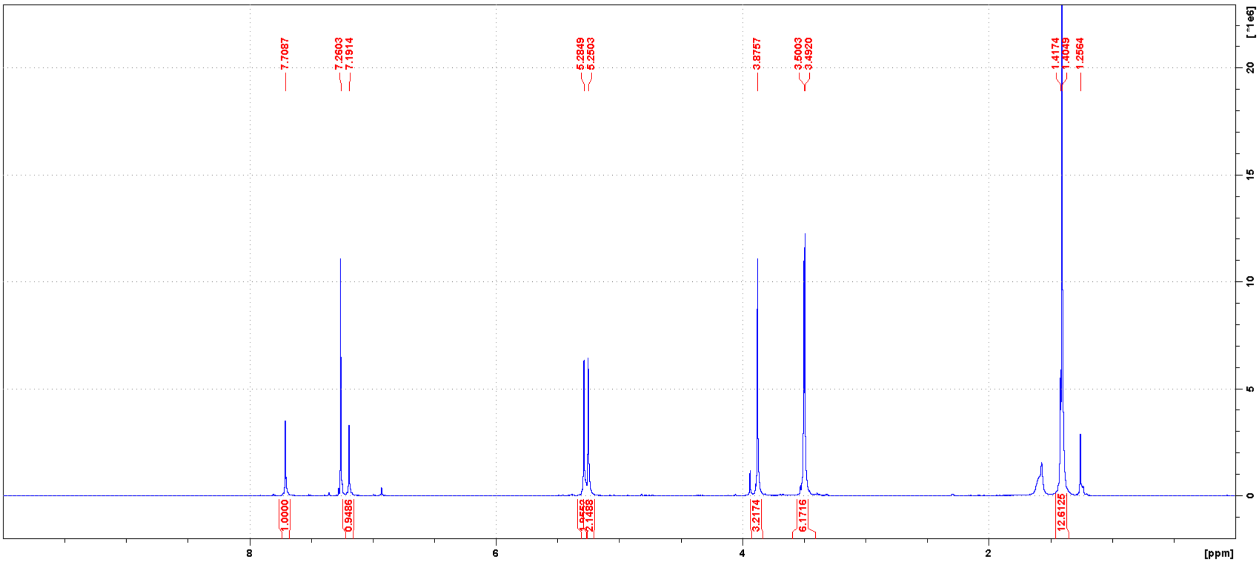

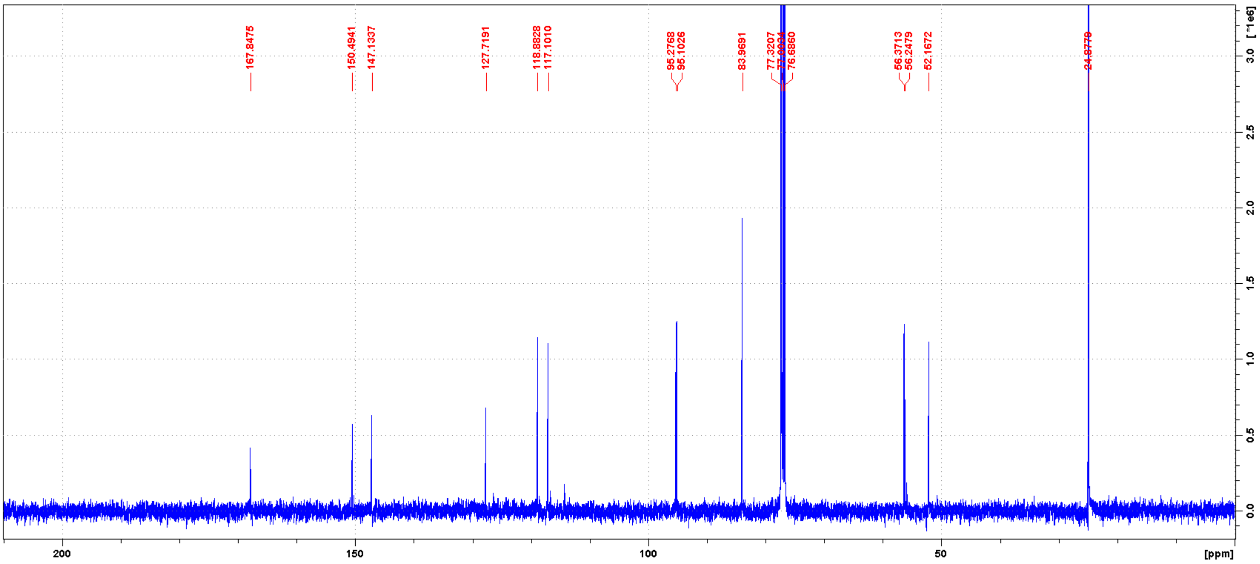

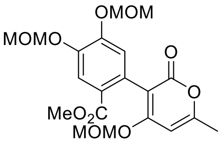
OMOM
MOMO
O
O
MeO2C
MOMO

16

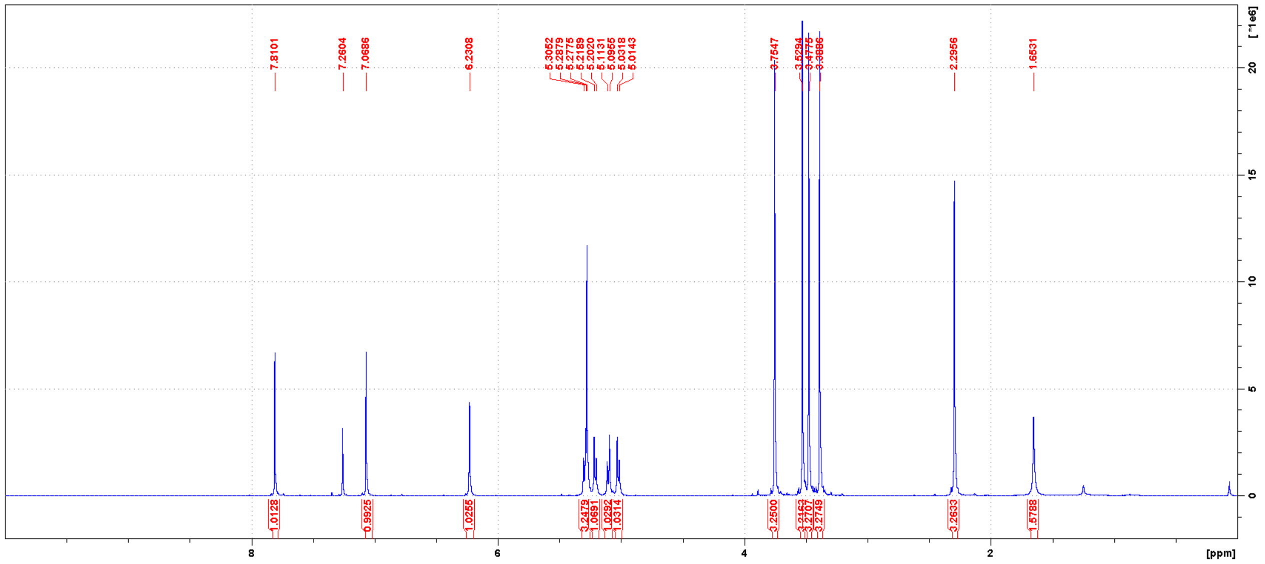
7.8101
7.2604
7.0686
6.2308
3.7547
2.2956
1.6531
1.0128
0.9925
1.0255
3.2500
3.2633
1.5788
[ppm]
[*1e6]

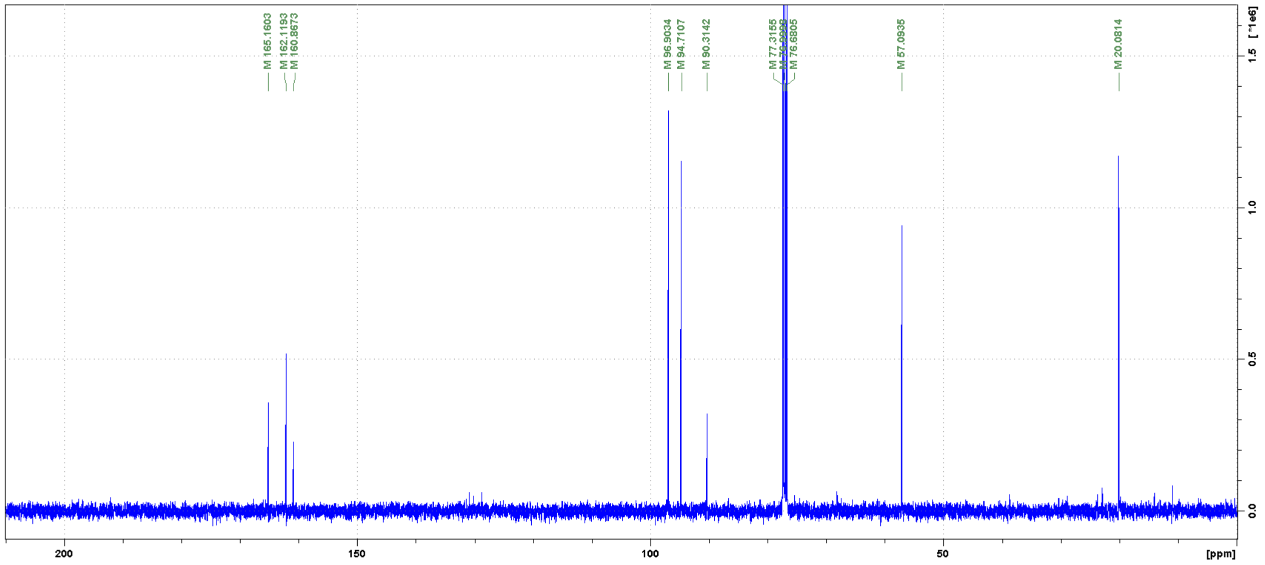
M 165.1603
M 162.1193
M 160.8673
M 96.9034
M 94.7107
M 90.3142
M 77.3156
M 76.6805
M 57.0935
M 20.0814
[ppm]
[*1e6]

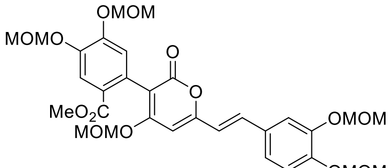

18

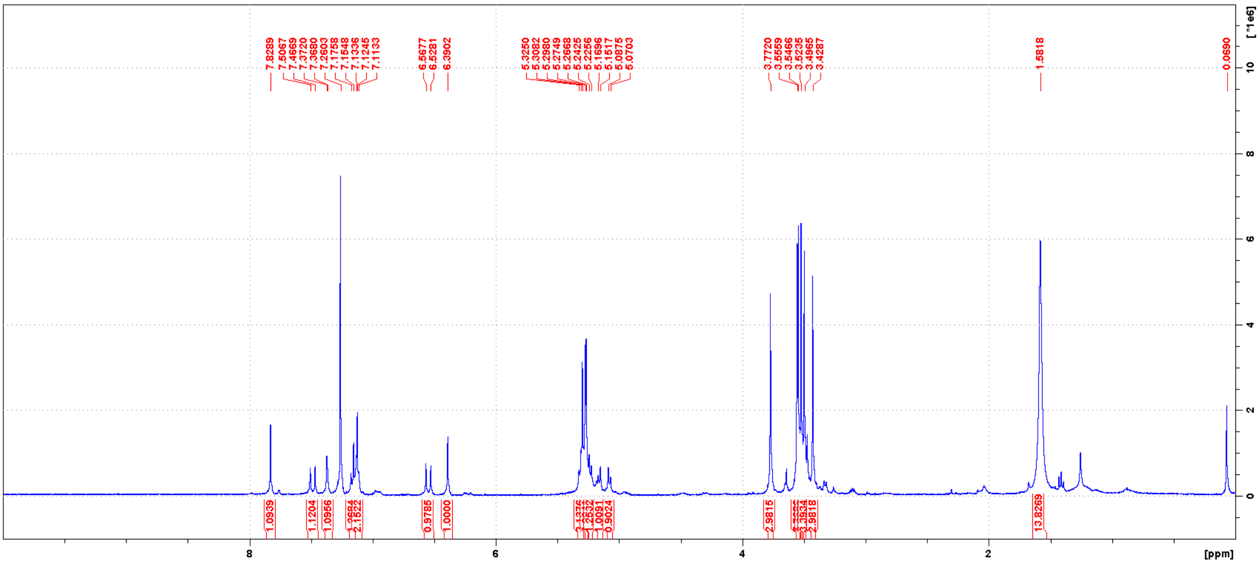

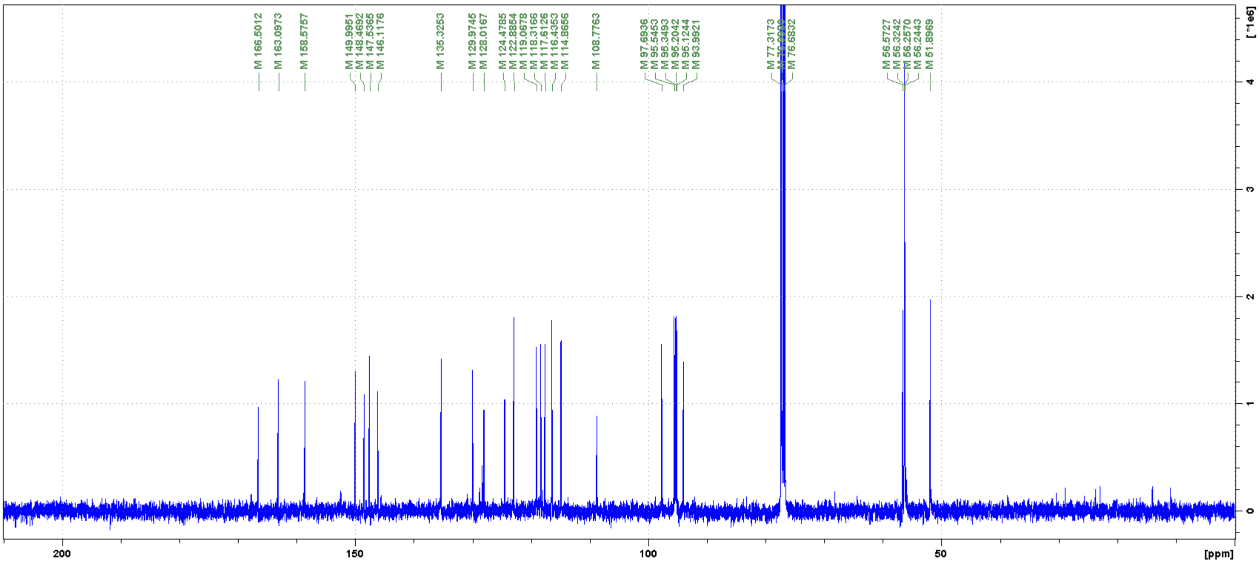

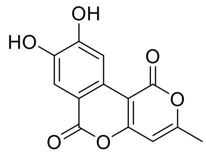

phelligridin A (**1**)

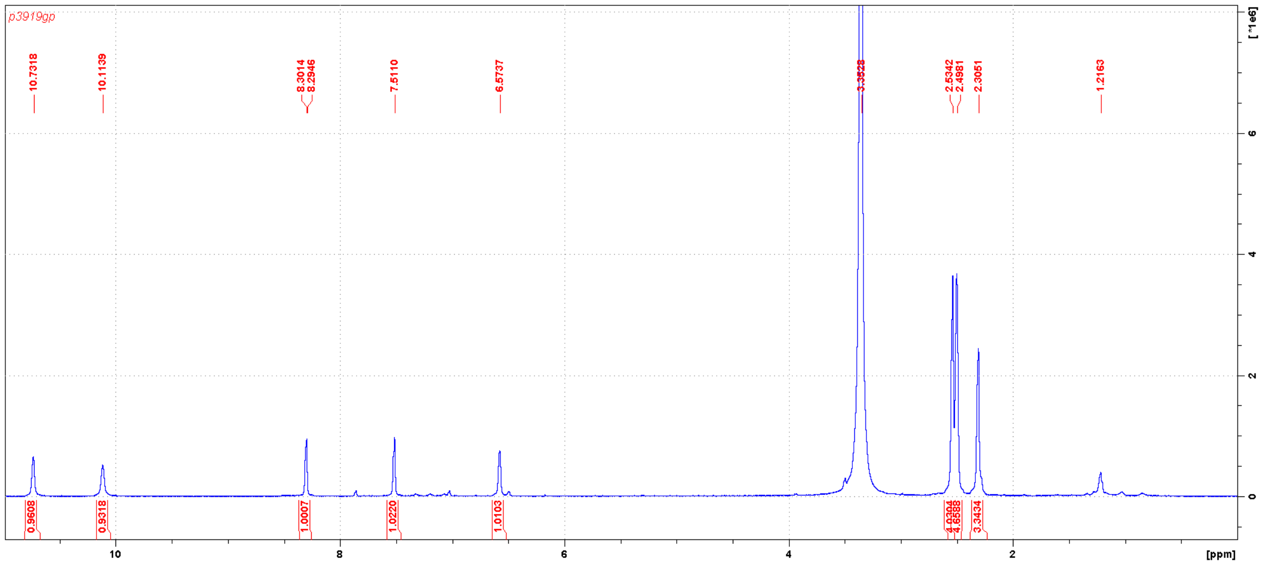

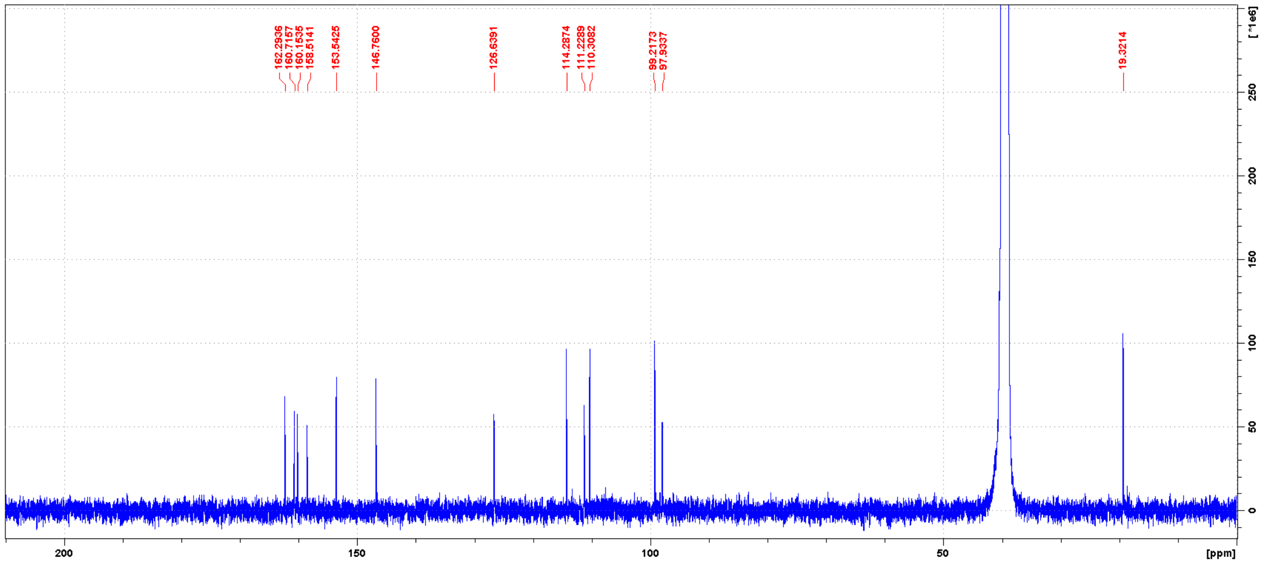

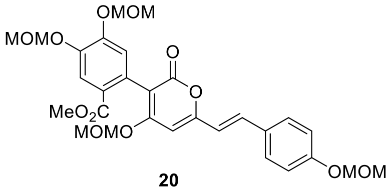

20

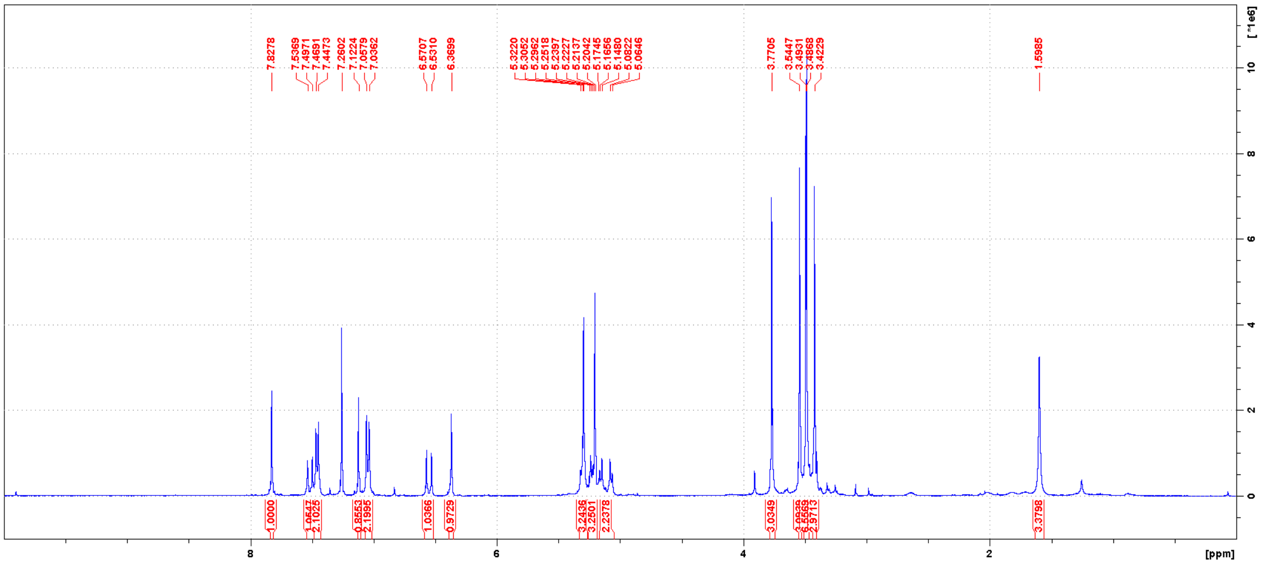

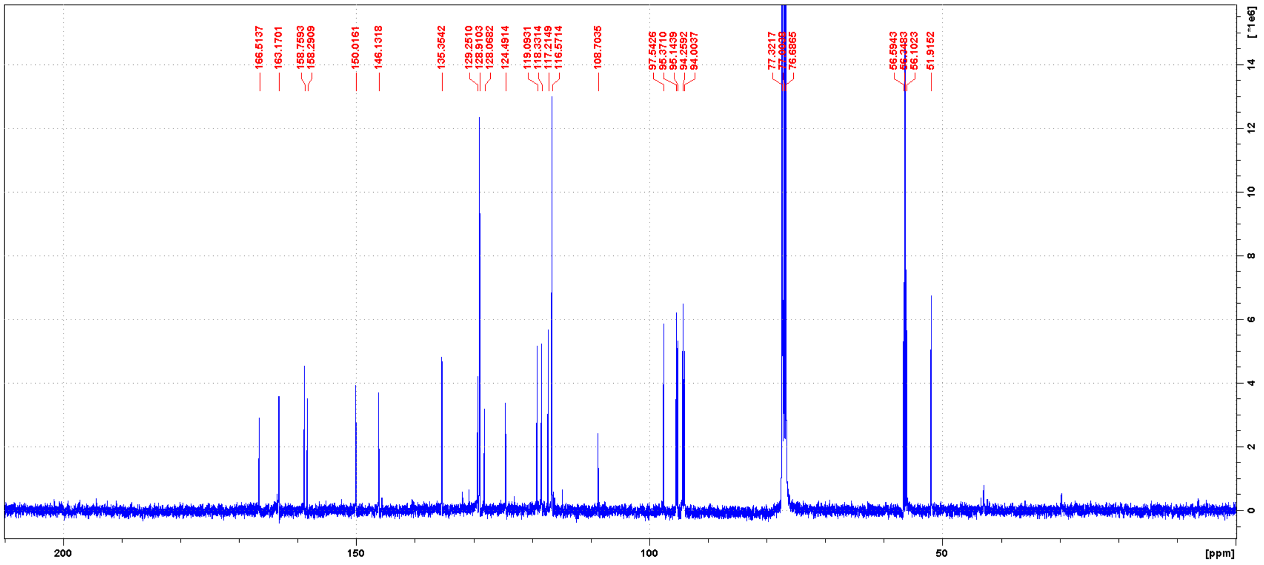

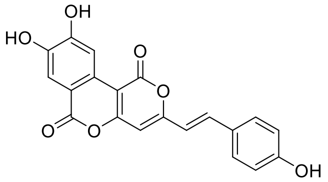

phelligridin C (**3**)

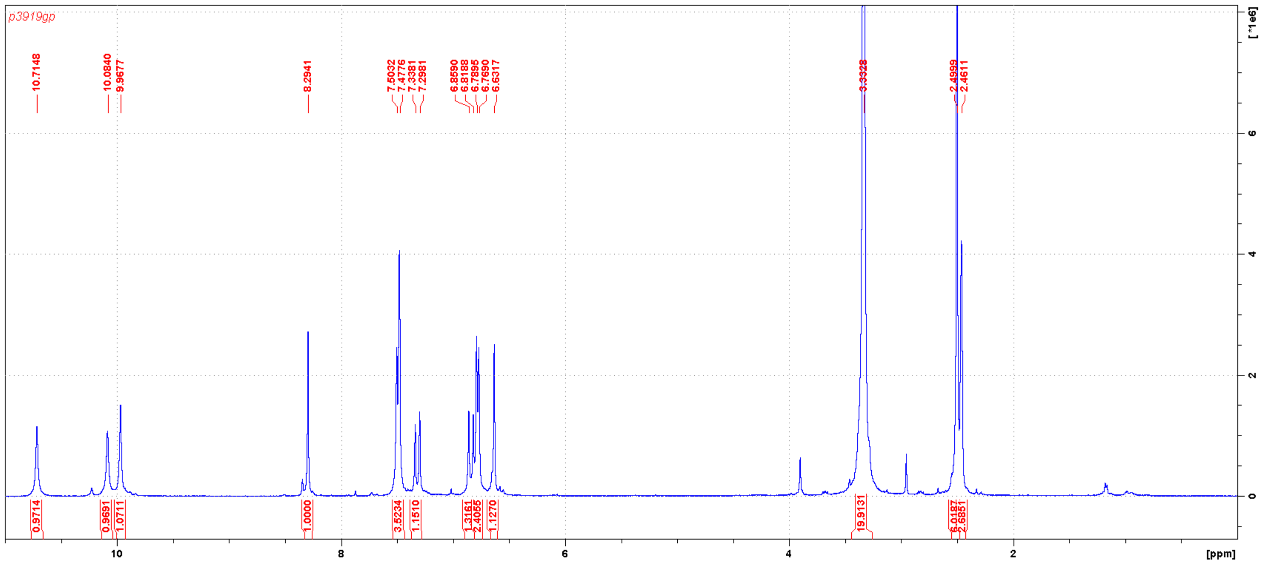

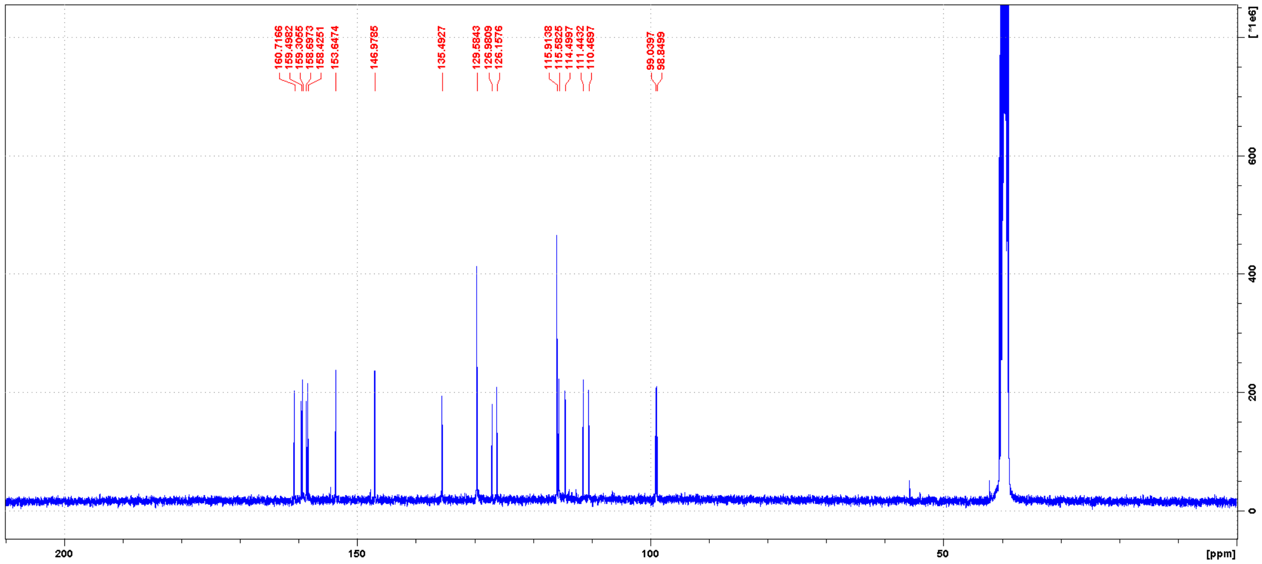

Supplement: RA-009-C8RA10346A-s001 [file RA-009-C8RA10346A-s001.pdf]
